# High Humidity Exacerbates Rheumatoid Arthritis in Mice via *Prevotella stercorea*-Mediated Chondroitin Sulfate Degradation

**DOI:** 10.3390/microorganisms14071540

**Published:** 2026-07-14

**Authors:** Mingzhu Wang, Qianqian He, Yiwu Qiu, Lin Huang, Yun Zhang, Ding Ye, Zhixing He, Chengping Wen

**Affiliations:** 1 Research Institute of Chinese Medical Clinical Foundation and Immunology, School of Basic Medical Science, Zhejiang Chinese Medical University, Hangzhou 310053, China; 2 Department of Epidemiology, School of Public Health, Zhejiang Chinese Medical University, Hangzhou 310053, China

**Keywords:** rheumatoid arthritis, humidity, collagen-induced arthritis, *Prevotella stercorea*, glycosaminoglycan, chondroitin sulfate

## Abstract

**Background:** Rheumatoid arthritis (RA) is influenced by environmental exposures. High humidity has been clinically associated with worsened joint symptoms, but the microbial and metabolic mechanisms remain unclear. We investigated whether a gut microbiota–metabolism axis contributes to humidity-associated aggravation of collagen-induced arthritis (CIA). **Methods:** CIA mice were maintained under normal or high relative humidity. We integrated 16S rRNA and metagenomic sequencing, liquid chromatography–tandem mass spectrometry metabolomics, and intestinal barrier assessments. Fecal microbiota transplantation (FMT) was performed to evaluate microbiota dependency. Based on multi-omics findings, we quantified chondroitin sulfate (CS) and conducted functional experiments involving *Prevotella stercorea* (*P. stercorea*) supplementation, CS administration, and in vitro degradation assays. **Results:** High humidity aggravated arthritis severity and systemic inflammation, including increased interleukin-6, interleukin-17A, and granulocyte colony-stimulating factor, and was accompanied by impaired intestinal barrier integrity. FMT supported a microbiota-dependent contribution. Metagenomic analysis identified enrichment of *P. stercorea* and glycosaminoglycan degradation pathways under high humidity. CS abundance was reduced in articular cartilage, *P. stercorea* degraded CS in vitro and was associated with cartilage CS loss in vivo, and CS supplementation attenuated arthritis under high humidity and reduced the arthritis-promoting effects associated with *P. stercorea*. **Conclusions:** High humidity is associated with microbiota-dependent functional remodeling, enhanced CS degradation, and aggravated arthritis in CIA mice. These findings suggest that humidity-associated alterations in microbial CS metabolism may link environmental exposure to cartilage disruption and joint inflammation.

## 1. Introduction

Rheumatoid arthritis (RA) is a chronic immune-mediated inflammatory disease characterized by persistent synovitis, progressive cartilage destruction, and irreversible joint dysfunction [1]. Globally, RA affects 0.3~1% of the population and is increasingly recognized as a significant public health challenge [2]. RA progression causes joint erosion, functional impairment, and a decrease in overall quality of life [3]. Although genetic susceptibility and immune dysregulation are central to RA pathogenesis, accumulating evidence suggests that disease onset and progression are profoundly shaped by environmental exposures [4,5,6].

Environmental humidity has long been recognized as a clinically relevant factor associated with arthritis symptoms [7,8]. Patients with RA frequently report symptom exacerbation in humid environments, and epidemiological studies have linked higher ambient humidity to increased disease activity and inflammatory burden. Despite these observations, environmental humidity has largely been regarded as a nonspecific trigger rather than a mechanistically relevant exposure [9,10,11].

Emerging concepts in translational immunology emphasize the gut microbiota as a central interface linking environmental exposures to host immune responses. The gut microbiota not only responds dynamically to environmental changes but also regulates systemic immunity through microbial metabolites, barrier integrity, and immune cell programming [12]. Dysbiosis has been consistently reported in RA patients and animal models, and alterations in gut microbial composition can precede clinical joint manifestations, suggesting a causal role in disease development [13,14,15,16,17,18]. Notably, antibiotic-induced depletion of gut microbiota has been shown to alleviate joint swelling and reduce the severity of arthritis, highlighting the critical role of gut microbiota in RA [19]. Importantly, environmental factors such as diet, temperature, and humidity are increasingly recognized as key drivers of microbiota functional remodeling rather than merely changes in microbial abundance.

Recent studies have shown that high humidity can alter gut microbial composition and metabolic activity in autoimmune and metabolic disease settings [20,21,22,23,24]. These observations raise the possibility that humidity-induced functional remodeling of the microbiota contributes to immune-mediated joint inflammation.

Glycosaminoglycans are major structural components of articular cartilage. Chondroitin sulfate (CS) contributes to extracellular matrix integrity and modulates inflammatory signaling, whereas excessive glycosaminoglycan degradation is associated with cartilage erosion. Because several gut microorganisms possess glycosaminoglycan-degrading functions, humidity-dependent microbial metabolism could provide a mechanistic link between environmental exposure and cartilage homeostasis. Among the arthritis-associated gut microbes, members of the genus *Prevotella* have attracted attention because of their reported associations with RA susceptibility and intestinal immune activation; however, whether specific *Prevotella* species participate in humidity-associated microbial glycosaminoglycan metabolism remains unclear.

Taken together, these findings reveal several important knowledge gaps. First, although high humidity has been clinically associated with aggravated arthritis symptoms, the biological mechanisms by which humidity amplifies joint inflammation remain poorly understood. Second, while the gut microbiota is increasingly recognized as a key interface between environmental exposures and systemic immunity, whether humidity-induced microbial remodeling directly contributes to immune-mediated arthritis has not been established. Third, although glycosaminoglycan degradation and CS loss are closely related to cartilage destruction, it remains unknown whether environmental humidity promotes arthritis progression by reshaping microbial glycosaminoglycan metabolism.

In this study, we hypothesized that environmental humidity acts as a modifiable exposure that exacerbates arthritis by reprogramming gut microbiota function and disrupting host cartilage metabolism. Using a collagen-induced arthritis (CIA) mouse model, we sought to determine whether high humidity aggravates arthritis through a gut microbiota-dependent mechanism and to identify the microbial and metabolic pathways underlying this process. By integrating metagenomic sequencing, fecal metabolomics, fecal microbiota transplantation (FMT), and targeted microbial and metabolic interventions, we further aimed to determine whether humidity-associated microbial glycosaminoglycan metabolism links gut microbial remodeling to cartilage metabolic disruption and inflammatory amplification. Candidate microbial taxa, including *Prevotella stercorea* (*P. stercorea*), were then prioritized for functional validation based on metagenomic evidence, including humidity-dependent abundance patterns and pathway contributions.

## 2. Methods

### 2.1. Animals and the Construction of the CIA

Three-week-old male DBA/1 mice were purchased from Shanghai SLAC Laboratory Animal Co., Ltd. (Shanghai, China). After arrival, the mice underwent a one-week acclimatization period before randomization [25]. They were housed under a 12 h/12 h light/dark cycle at 25 ± 1 °C with free access to food and water. Relative humidity was controlled using a climate chamber (RXZ-380A, Ningbo Jiangnan Instrument Factory, Ningbo, China), and recorded temperature and humidity profiles are provided in Figure S1. All mice were maintained under the same standard husbandry conditions throughout the study and received the same standard irradiated laboratory mouse growth and reproduction diet (CRO; Jiangsu Xietong Pharmaceutical Bioengineering Co., Ltd., Nanjing, China; Cat. No. XTI01CR-010). The ingredient list and nutritional composition of the diet are provided in Supplementary Table S1. All animal procedures were approved by the Ethics Committee of Zhejiang Chinese Medical University (Approval No. 20210816-12).

To establish the CIA model, the mice were injected with 200 μg of bovine type II collagen (Chondrex, Redmond, WA, USA) in 200 μL of complete Freund’s adjuvant on day 21, followed by an injection of the same dose in incomplete Freund’s adjuvant on day 42. All animal experiments were conducted following the ethical guidelines and regulations set by the Institutional Animal Care and Use Committee of Zhejiang Chinese Medical University, in accordance with the institution’s Guidelines for Animal Experimentation.

### 2.2. Culture Conditions of P. stercorea

*P. stercorea* (DSM 18206) [26] was obtained from the DSMZ (Braunschweig, Germany). The strain was routinely cultivated in Fastidious Anaerobe Broth (FAB; ELITE-MEDIA, Cat. No. M260-01) at 37 °C for 48 h under anaerobic conditions. To quantify the bacterial dose, aliquots of the suspension were plated on sheep blood agar and incubated anaerobically at 37 °C for 48 h before colony-forming units (CFU) were counted. To assess CS degradation, *P. stercorea* was cultivated in minimal salt medium (MSM; Hopebio, Qingdao, China, Cat. No. HB9214) supplemented with CS at 5, 10, or 15 mg/mL as the sole carbon source. MSM was prepared according to the manufacturer’s instructions (4.403 g/L distilled water, sterilized at 121 °C for 15 min), and filter-sterilized CS was added at the indicated concentrations.

### 2.3. Animal Experimental Design

Two relative humidity conditions were used: 50% (normal humidity) and 80% (high humidity), both at 25 ± 1 °C. The 80% condition was selected from previous studies identifying this range as high humidity [27,28] and from our preliminary observations, in which reductions in food and water intake occurred at 90% but not at 80% relative humidity [6]. To further characterize the environmental exposure conditions, the temperature-humidity index (THI) was calculated using the formula: THI = T − (0.55 − 0.0055 × RH) × (T − 14.5), where T represents ambient temperature in °C and RH represents relative humidity (%) [29]. Under the controlled temperature condition of 25 ± 1 °C, the estimated THI was 22.11 for the normal-humidity group (50% RH) and 23.85 for the high-humidity group (80% RH).

### 2.4. Experiment I: Effect of High Humidity on Arthritis in CIA Mice

**Figure 1:**
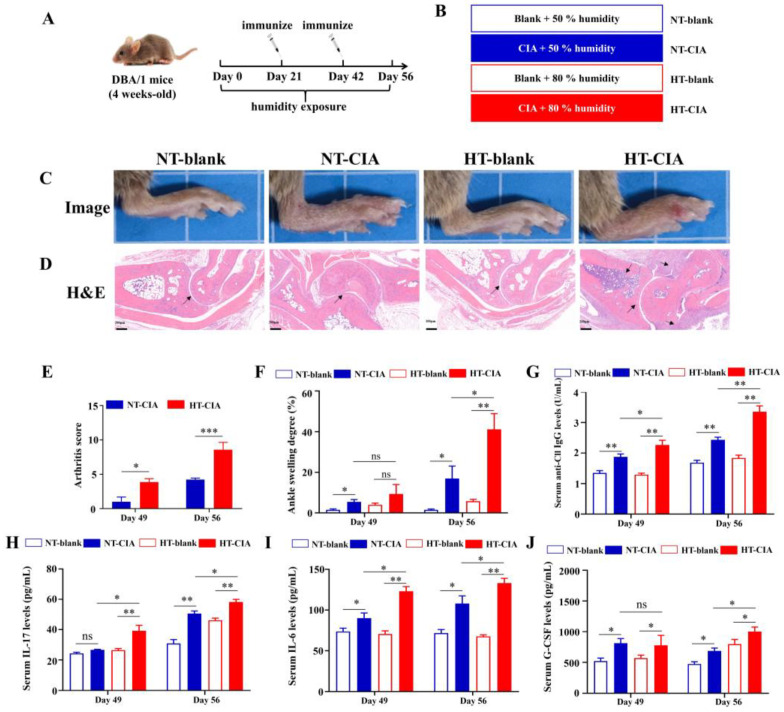
Effect of high humidity on arthritis symptoms in CIA mice. (**A**) Overview of study design. 4-week-old DBA/1 mice were subjected to CIA modeling with immunization on Day 21 and Day 42, and exposed to different humidity conditions from Day 0 to Day 56. (**B**) Group assignment: NT-blank (blank control with normal humidity, 50%); NT-CIA (CIA model with normal humidity, 50%); HT-blank (blank control with high humidity, 80%); HT-CIA (CIA model with high humidity, 80%). (**C**) Representative images showing ankle swelling in each group. (**D**) Representative H&E-stained histological sections of ankle joints, with arrows indicating inflammatory cell infiltration and synovial hyperplasia. (**E**) Arthritis scores in NT-CIA and HT-CIA groups on Day 49 and Day 56. (**F**) Ankle swelling degrees in each group on Day 49 and Day 56. (**G**–**J**) Serum levels of anti-CII IgG, IL-17, IL-6, and G-CSF in each group on Day 49 and Day 56, detected by enzyme-linked immunosorbent assay (ELISA). Values are presented as the mean ± SEM. “***” denotes *p* < 0.001, “**” denotes *p* < 0.01, “*” denotes *p* < 0.05, “ns” denotes *p* > 0.05. Abbreviations used: CIA, collagen-induced arthritis; NT, normal humidity (50%); HT, high humidity (80%).

DBA/1 mice were randomly assigned to normal humidity (NT) or high humidity (HT) conditions. After 21 days, each humidity group was divided into a blank subgroup, which received 0.9% NaCl on days 21 and 42, and a CIA subgroup, which received bovine type II collagen on days 21 and 42. The study design and group allocation are summarized in Figure 1A,B.

### 2.5. Experiment II: Effect of FMT on Arthritis in CIA Mice

The FMT experiment was conducted in parallel with Experiment I. CIA mice received broad-spectrum antibiotics (ampicillin 0.2 g/L, vancomycin 0.1 g/L, neomycin 0.2 g/L, and metronidazole 0.2 g/L) by oral gavage at 200 μL/day from day 22 to day 24. This short-term antibiotic pretreatment was selected based on previously reported antibiotic-based microbiota-depletion protocols in mice [30]. From day 25 to day 56, mice received 200 μL/day of freshly prepared fecal suspension. Fresh feces were resuspended in 0.9% saline at a 5:1 weight-to-volume ratio, filtered through a 20 μm filter, and administered on the day of collection.

### 2.6. Experiment III: Effect of Prevotella Stercorea on Arthritis in CIA Mice

For the *P. stercorea* supplementation experiment, mice were assigned to blank, CIA, or CIA + *P. stercorea* groups under each humidity condition. CIA was induced on days 21 and 42. The supplementation group received the broad-spectrum antibiotic mixture by oral gavage at 200 μL/day from day 22 to day 24, followed by live *P. stercorea* at 3 × 10^7^ CFU/mouse/day from day 25 to day 56. The dose was selected from previously reported *Prevotella* colonization ranges in murine arthritis studies [31,32].

### 2.7. Experiment IV: Effect of Chondroitin Sulfate on Arthritis in CIA Mice

For the CS supplementation experiment, mice under each humidity condition were assigned to blank, CIA, or CIA + CS groups. Blank mice received 0.9% NaCl and sterile water, CIA mice received collagen and sterile water, and CIA + CS mice received collagen plus CS (150 mg/kg/day; MCE, catalog no. HY-B2162) by oral gavage from day 21 to day 56 [33]. The oral route was selected because it matches the cited intervention protocol and permits evaluation of effects involving the intestinal microbial environment.

### 2.8. Experiment V: Effects of Concurrent Chondroitin Sulfate and Prevotella stercorea Treatment on Arthritis in CIA Mice

For the combined intervention experiment, mice exposed to high humidity were assigned to blank, CIA, CIA + CS, CIA + *P. stercorea*, or CIA + CS + *P. stercorea* groups. CS was administered orally at 150 mg/kg/day from day 21 to day 56. Groups receiving *P. stercorea* were given the broad-spectrum antibiotic mixture at 200 μL/day from day 22 to day 24 and then live *P. stercorea* at 3 × 10^7^ CFU/mouse/day from day 25 to day 56. Control groups received the corresponding vehicle.

Notably, DBA/1 mice typically present arthritis symptoms between days 26 and 35 following the initial injection of type II collagen. Therefore, sampling was conducted on days 49 and 56, corresponding to 27 and 35 days post-injection, respectively.

### 2.9. Assessment of Arthritis Symptoms

Arthritis symptoms were evaluated using a modified version of the method described by Wang et al. [6]. After the second collagen injection, arthritis scores and ankle swelling were assessed every other day. Scoring was based on the extent of swelling and redness in the foot joints, with scores ranging from 0 (no symptoms) to 4 (severe swelling and redness in all paws). The maximum score of each mouse was 16. Ankle swelling was measured using a vernier caliper (DEGUQMNT, Suhl, Germany).

### 2.10. Measurement of Autoantibodies and Pro-Inflammatory Cytokines

Blood samples were collected from the orbital vein on days 42 and 56 and centrifuged at 3000 rpm for 15 min at 4 °C to obtain serum. Serum type II collagen antibody (anti-CII IgG, CUSABIO, Wuhan, China, CAS: CSB-EQ027975MO) levels were quantified using CUSABIO ELISA Kits based on the double antigen sandwich ELISA method. Additionally, serum inflammatory cytokines, including interleukin-6 (IL-6, Multisciences, Hangzhou, China, CAS: EK206/3-96), interleukin-17A (IL-17A, CAS: EK217HS-96), and granulocyte colony stimulating factor (G-CSF, Multisciences, Hangzhou, China, CAS: EK269/2-96), were measured using Multisciences ELISA Kits following the methods outlined in our previous research [6].

### 2.11. Histopathological Investigations

Freshly collected ankle tissues were fixed in 4% paraformaldehyde, dehydrated in ethanol, cleared in xylene, embedded in paraffin wax at 56 °C, and sectioned into 5 μm-thick slices. All tissue sections were stained with hematoxylin and eosin (H&E) for histopathological analysis. Images were captured using an optical microscope (NIKON ECLIPSE E100, Tokyo, Japan) equipped with an imaging system (NIKON DS-U3, Tokyo, Japan). Finally, the morphology and histological scores were analyzed using CaseViewer software 2.4 (3DHISTECH CaseViewer, Budapest, Hungary).

### 2.12. Metagenomic Sequencing and Analysis

Following euthanization, stool samples were collected from the colon and immediately preserved at −80 °C for subsequent analysis. Genomic DNA was extracted from the stool samples using the QIAamp Fast DNA Stool Mini Kit (Qiagen, Hilden, Germany, CAS: 51604) according to the manufacturer’s instructions. Shotgun metagenomic sequencing of fecal genomic DNA was conducted by Majorbio Bio-Pharm Technology Co., Ltd., Shanghai, China. Further details are described in our previous publication [34]. For each sample, a paired-end library with an average size of 400 bp was constructed and sequenced using Illumina NovaSeq (Illumina Inc., San Diego, CA, USA). The raw sequence data associated with this project has been deposited in the NCBI Short Read Archive database (Accession Number: PRJNA917043).

The raw reads from metagenome sequencing were analyzed using the Majorbio Cloud Platform (www.majorbio.com). After filtering and quality control, high-quality sequences were obtained as previously reported [34]. Representative sequences from the non-redundant gene catalog were aligned to the NR database using Diamond with an e-value cutoff of 1 × 10^−5^ to annotate taxonomic information. Kyoto encyclopedia of genes and genomes (KEGG) annotation was performed by aligning sequences to the Kyoto Encyclopedia of Genes and Genomes database (http://www.genome.jp/kegg/, accessed on 20 May 2024) using Diamond with a similar e-value cutoff of 1 × 10^−5^. To compare the taxonomic and KEGG pathway differences between groups, the linear discriminant analysis (LDA) effect size (LEfSe) method was employed, with a logarithmic LDA score threshold of 2.0 to determine significance.

### 2.13. Metabolomics Analysis of Colonic Feces

Colonic feces samples were prepared for liquid chromatography-tandem mass spectrometry (LC-MS/MS) analysis following our previously established method [35]. The analysis was conducted by Majorbio Bio-Pharm Technology Co., Ltd. (Shanghai, China) using a UHPLC-Q Exactive system from Thermo Fisher Scientific. The chromatographic and mass spectrometric conditions were similar to those described in our prior study [35]. After completing the mass spectrometry detection, the raw LC/MS data were preprocessed using Progenesis QI software v2.4 (Waters Corporation, Milford, MA, USA). A three-dimensional data matrix in CSV format was exported, containing sample information, metabolite names, and mass spectral response intensities. Internal standard peaks and false positive peaks, including noise, column bleed, and derivatized reagent peaks, were removed from the data matrix. Subsequently, de-redundancy and peak pooling were performed. Metabolites were identified using the HMDB (http://www.hmdb.ca/, accessed on 25 May 2024), Metlin (https://metlin.scripps.edu/), and Majorbio databases.

The processed data were uploaded to the Majorbio cloud platform (https://cloud.majorbio.com) for analysis. Metabolic features detected in at least 80% of any set of samples were retained. For specific samples where metabolite levels fell below the lower limit of quantitation, minimum metabolite values were imputed, and each metabolic feature was normalized by sum normalization to reduce errors from sample preparation and instrument instability.

The R package ropls (Version 1.6.2) was used for orthogonal partial least squares discriminant analysis (OPLS-DA), incorporating 7-cycle interactive validation to assess model stability. Additionally, Student’s *t*-test and fold difference analysis were performed. Significantly different metabolites were identified based on the Variable Importance in Projection (VIP) scores from the OPLS-DA model and the *p*-values from the student’s *t*-test, using thresholds of VIP > 1 and *p* < 0.05. Differential metabolites between groups were summarized and mapped into biochemical pathways through metabolic enrichment and pathway analysis using the KEGG database (http://www.genome.jp/kegg/, accessed on 25 May 2024). These metabolites were classified according to their relevant pathways or functions.

### 2.14. Determination of Chondroitin Sulfate

CS abundance in articular cartilage was assessed by immunofluorescence staining. Joint sections were blocked with 3% bovine serum albumin for 30 min at room temperature and incubated overnight at 4 °C with an anti-chondroitin sulfate antibody (1:2500; Abcam, Waltham, MA, USA). Sections were then incubated with the appropriate fluorescence-conjugated secondary antibody (1:500; Abcam) at 37 °C for 50 min in the dark, counterstained with DAPI, washed with phosphate-buffered saline, treated with autofluorescence quencher, mounted, and imaged by fluorescence microscopy. Fluorescence intensity was used as a relative measure of tissue CS abundance rather than an absolute concentration.

Fecal CS was identified from the LC–MS/MS-based fecal metabolomics dataset described in Section 2.13 and was presented as relative abundance.

Residual CS in minimal salt medium was measured by HPLC using a Shimadzu LC-20AD system (Shimadzu, Kyoto, Japan) coupled to an SPD-M20A photodiode array detector. Derivatized products were separated on a Diamonsil-Plus C18 column (4.6 × 250 mm, 5 μm) at 35 °C using 5 mmol/L sodium heptanesulfonate-acetonitrile (95:5, *v*/*v*) at 0.6 mL/min.

### 2.15. Statistical Analysis

Statistical analyses were performed using GraphPad Prism version 8.0 (GraphPad Software Inc., San Diego, CA, USA). Data are presented as the mean ± SEM. For comparisons involving more than two groups or multiple prespecified pairwise comparisons, one-way or two-way ANOVA was used as appropriate, followed by Holm–Šídák multiple-comparison correction. Multiple-comparison correction was applied within each figure or outcome family. Exact raw *p* values and adjusted *p* values for the major prespecified comparisons are provided in Supplementary Table S2 where applicable. Statistical significance was defined as adjusted *p* < 0.05 where multiple-comparison correction was applied. For metabolomic analyses, differential metabolites were identified based on variable importance in projection (VIP) > 1 and *p* < 0.05, as described above.

## 3. Results

### 3.1. High Environmental Humidity Exacerbates Arthritis Severity in CIA Mice

As shown in Figure 1, immunization with type II collagen to induced arthritis manifestations in DBA/1 mice (Figure 1A,B), including ankle swelling, synovial inflammation, joint destruction, and increased serum anti-CII IgG levels, under both normal and high humidity conditions (Figure 1C–G).

Comparison between humidity conditions revealed that exposure to high humidity was associated with more severe arthritis outcomes. CIA mice housed under high humidity displayed increased inflammatory cell infiltration in ankle joints, higher arthritis scores, greater ankle swelling, and elevated serum anti-CII IgG levels on days 49 and 56 compared with mice maintained under normal humidity (Figure 1C–G and Figure S2A, *p* < 0.05).

Given the systemic inflammatory nature of RA, serum proinflammatory cytokines were further assessed. On day 49, CIA mice exposed to high humidity exhibited significantly increased serum levels of IL-6, IL-17A, and G-CSF, whereas mice under normal humidity showed elevated IL-6 and G-CSF levels (Figure 1H–J, *p* < 0.05). By day 56, serum IL-6, IL-17A, and G-CSF levels were increased in CIA mice under both humidity conditions. Notably, mice exposed to high humidity demonstrated higher serum IL-6 and IL-17A levels on days 49 and 56, as well as increased G-CSF levels on day 56, compared with those maintained under normal humidity (Figure 1H–J, *p* < 0.05).

### 3.2. High Humidity Is Associated with Alterations in Gut Microbiota and Intestinal Barrier Integrity in CIA Mice

To determine whether the aggravation of arthritis under high humidity was accompanied by alterations in the gut microenvironment, we examined gut microbiota composition and intestinal barrier integrity in CIA mice exposed to different humidity conditions.

**Figure 2:**
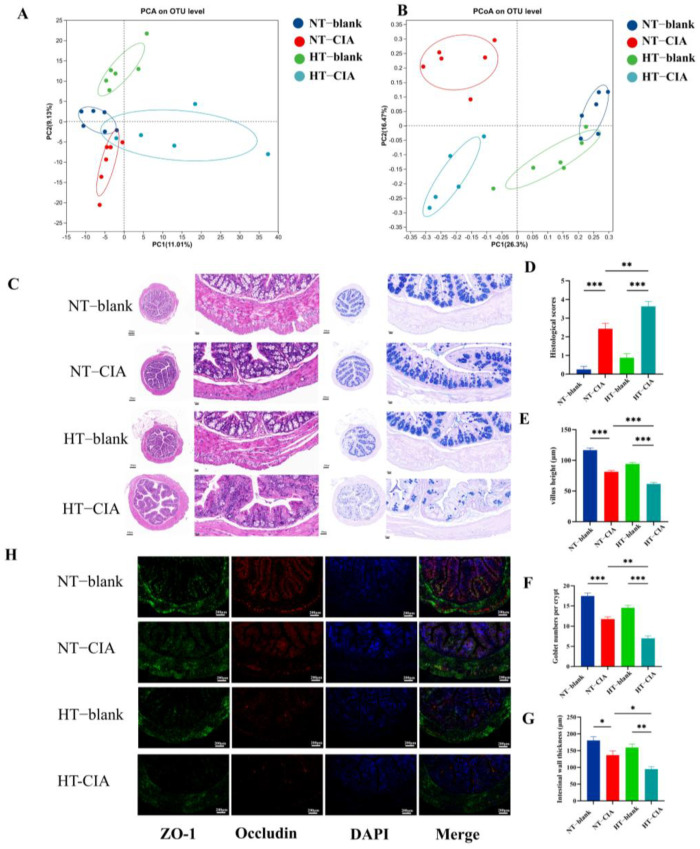
Effect of high humidity on gut microbiota composition and intestinal barrier integrity in CIA mice. (**A**) Principal component analysis (PCA) score plot of gut microbiota composition at the OTU level across all groups. (**B**) Principal coordinate analysis (PCoA) plot of gut microbiota composition at the OTU level across all groups. (**C**) Representative images of H&E and Alcian blue-periodic acid-Schiff (AB-PAS) stained colon tissue sections. (**D**) Histological scores of colon tissue in each group. (**E**) Villus height of colon tissue in each group. (**F**) Goblet cell numbers in colon tissue in each group. (**G**) Intestinal wall thickness of colon tissue in each group. (**H**) Representative immunofluorescence images of the intestinal tight junction proteins ZO-1 (green) and Occludin (red), with DAPI (blue) nuclear counterstaining, in colon tissues from each group. Values are presented as the mean ± SEM. “***” denotes *p* < 0.001, “**” denotes *p* < 0.01, “*” denotes *p* < 0.05, “ns” denotes *p* > 0.05. Abbreviations used: FMT, fecal microbiota transplantation; CIA, collagen-induced arthritis; NT, normal humidity (50%); HT, high humidity (80%); PCA, principal components analysis; PCoA, principal coordinates analysis; OTU, operational taxonomic unit.

16S rRNA gene sequencing analysis showed that CIA mice housed under high humidity exhibited gut microbiota profiles distinct from those observed under normal humidity conditions. Principal coordinate analysis demonstrated a clear separation between the two groups, indicating that environmental humidity was associated with alterations in gut microbial community structure during arthritis progression (Figure 2A,B).

Consistently, histological examination showed increased epithelial disruption and inflammatory cell infiltration in the colons of CIA mice under high humidity conditions (Figure 2C–G; *p* < 0.05). In parallel, evaluation of intestinal barrier integrity revealed that CIA mice exposed to high humidity displayed reduced expression of the tight junction-associated proteins ZO-1 and occludin in colonic tissue compared with mice maintained under normal humidity (Figure 2H; *p* < 0.05).

In humidity-matched blank mice, the available baseline measures did not show the arthritis phenotype observed after collagen immunization; comparisons of blank groups are displayed in Figure 1 and Figure 2. In humidity-matched blank mice, 80% humidity alone produced only limited baseline changes compared with normal humidity and did not induce arthritis-like manifestations. HT-blank mice showed no ankle swelling or arthritis scores, and serum inflammatory markers remained substantially lower than those observed in HT-CIA mice. Although some gut-related indices showed mild changes under high humidity, these alterations were less pronounced than those observed after CIA induction. These findings suggest that 80% humidity alone was insufficient to reproduce the inflammatory and gut barrier phenotype observed in HT-CIA mice, but may create a permissive gut microenvironment that becomes pathologically relevant during arthritis progression. These observations distinguish the humidity-associated changes in CIA mice from baseline findings in healthy controls.

### 3.3. Gut Microbiota Mediates the Impact of High Humidity on Arthritis Severity in CIA Mice

**Figure 3:**
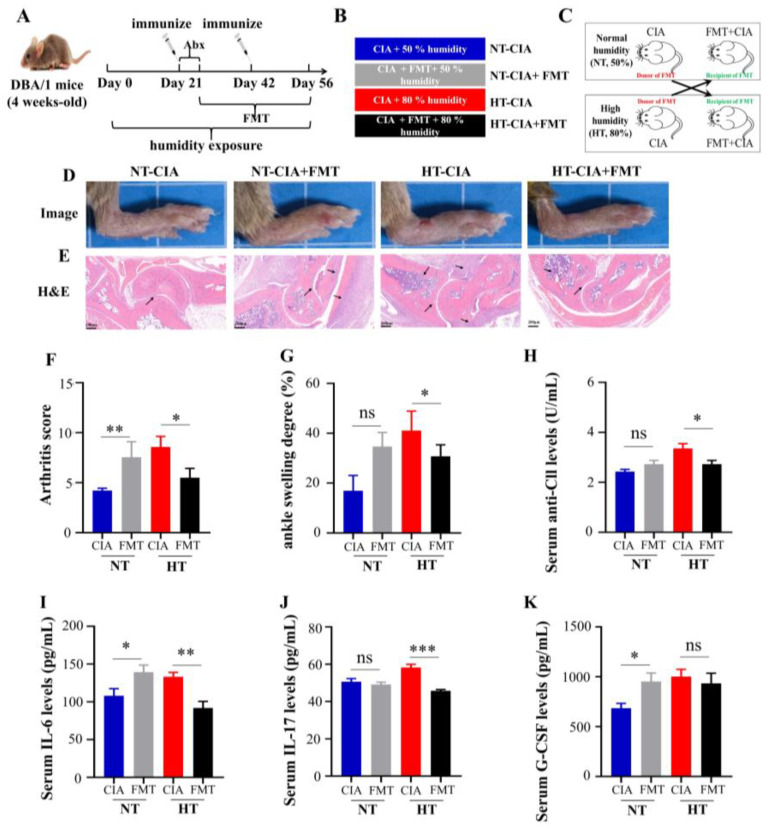
Effect of FMT on arthritis symptoms in humidity-adjusted CIA mice. (**A**) Overview of study design. 4-week-old DBA/1 mice were immunized on Day 21 and Day 42 for CIA modeling. Antibiotics (Abx) were administered at 200 μL/day from Day 22 to Day 24, followed by FMT exposure, and exposed to different humidity conditions from Day 0 to Day 56. (**B**) Group assignment: NT-CIA (CIA model with normal humidity, 50%); NT-CIA+FMT (CIA model with 50% humidity receiving FMT); HT-CIA (CIA model with high humidity, 80%); HT-CIA+FMT (CIA model with 80% humidity receiving FMT). (**C**) Schematic diagram illustrating the FMT strategy applied to CIA mice under NT and HT conditions. (**D**) Representative images showing ankle swelling in each group. (**E**) Representative H&E-stained histological sections of ankle joints, with arrows indicating inflammatory cell infiltration and synovial hyperplasia. (**F**) Arthritis scores in NT and HT groups with or without FMT treatment. (**G**) Ankle swelling degree (%) in NT and HT groups with or without FMT treatment. (**H**–**K**) Serum levels of anti-CII IgG, IL-6, IL-17, and G-CSF in each group detected by ELISA. Values are presented as the mean ± SEM. “***” denotes *p* < 0.001, “**” denotes *p* < 0.01, “*” denotes *p* < 0.05, “ns” denotes *p* > 0.05. Abbreviations used: FMT, fecal microbiota transplantation; CIA, collagen-induced arthritis; NT, normal humidity (50%); HT, high humidity (80%); Abx, antibiotics.

To determine whether the humidity-associated alterations in gut microbiota contribute to arthritis severity, FMT was performed between CIA mice housed under high and normal humidity conditions (Figure 3A–C).

Transplantation of gut microbiota from CIA mice exposed to high humidity into recipient CIA mice maintained under normal humidity resulted in more severe arthritis manifestations, including increased synovial cavity destruction and higher arthritis scores (Figure 3D–H and Figure S2B; *p* < 0.01). In contrast, transplantation of gut microbiota from CIA mice housed under normal humidity into recipients exposed to high humidity attenuated arthritis severity, as evidenced by reduced inflammatory cell infiltration in ankle joints, lower arthritis scores, decreased ankle swelling, and reduced serum anti-CII IgG levels on day 56 (Figure 3D–H; *p* < 0.05).

Consistent with these changes, FMT differentially affected systemic inflammatory responses depending on humidity conditions. In CIA mice under normal humidity, transplantation of microbiota derived from high-humidity donors was associated with increased serum levels of G-CSF and IL-6. Conversely, in CIA mice exposed to high humidity, transplantation of microbiota from normal-humidity donors was associated with reduced serum levels of IL-6 and IL-17A (Figure 3I–K; both *p* < 0.01).

These findings provide functional evidence that gut microbiota mediates the impact of high humidity on arthritis severity in CIA mice.

### 3.4. Metagenomic Analysis Identifies Humidity-Associated Enrichment of P. stercorea in CIA Mice

To further characterize gut microbial alterations associated with arthritis under different humidity conditions, metagenomic sequencing was performed to compare CIA mice with their respective humidity-matched control mice.

Under high humidity conditions, CIA mice exhibited 20 upregulated and 14 downregulated microbial species compared with humidity-matched control mice, accompanied by 7 upregulated and 2 downregulated KEGG pathways (Figures S3 and S4). In contrast, under normal humidity conditions, CIA mice displayed 54 upregulated and 19 downregulated species, along with 25 upregulated and 28 downregulated KEGG pathways, compared with normal-humidity control mice (Figures S5 and S6).

**Figure 4:**
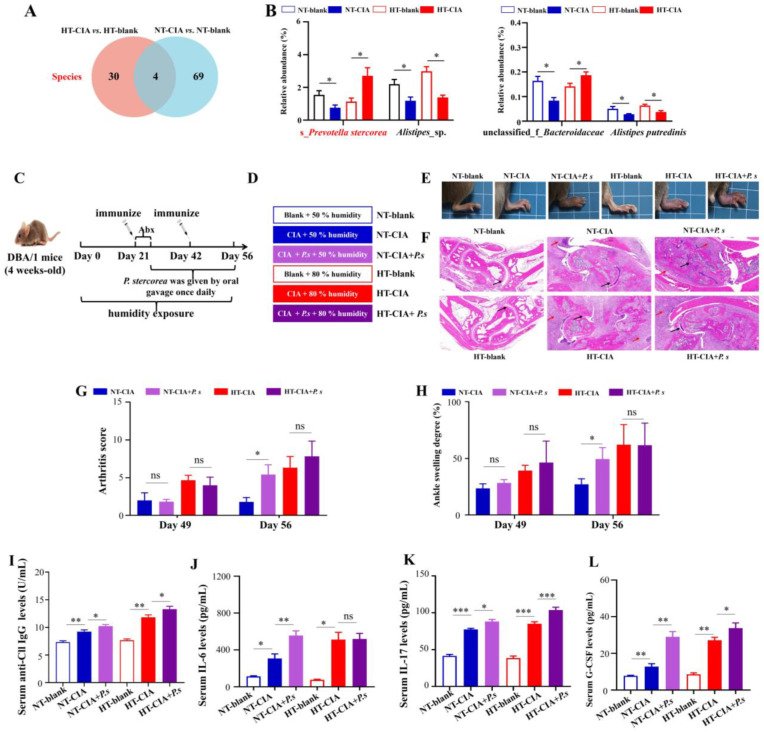
Humidity-associated enrichment of *P. stercorea* correlates with arthritis severity. (**A**) Venn diagram showing the overlap of differential bacterial species identified in the NT-blank vs. NT-CIA and HT-blank vs. HT-CIA comparisons. (**B**) Relative abundance of the key differential bacterial species (s_*P. stercorea*, *Alistipes* sp., unclassified_f_*Bacteroidaceae*, *Alistipes putredinis*) across all groups. (**C**) Overview of the study design for *P. stercorea* supplementation in CIA mice. 4-week-old DBA/1 mice were immunized on Day 21 and Day 42 for CIA modeling. Abx were administered at 200 μL/day from Day 22 to Day 24, then administered *P. stercorea* by oral gavage once daily, and exposed to different humidity conditions from Day 0 to Day 56. (**D**) Group assignment: NT-blank (blank control with normal humidity, 50%), NT-CIA (CIA model with normal humidity, 50%), NT-CIA+*P. stercorea* (CIA model with 50% humidity receiving *P. stercorea*), HT-blank (blank control with high humidity, 80%), HT-CIA (CIA model with high humidity, 80%), and HT-CIA+*P. stercorea* (CIA model with 80% humidity receiving *P. stercorea*). (**E**) Representative images showing ankle swelling in each group. (**F**) Representative H&E-stained histological sections of ankle joints. Black arrows indicate intact articular cartilage structure, and red arrows indicate inflammatory cell infiltration and synovial hyperplasia in joint tissues. (**G**) Analysis of arthritis scores in each group on Day 49 and Day 56. (**H**) Ankle swelling degree (%) in each group on Day 49 and Day 56. (**I**–**L**) Serum levels of anti-CII IgG, IL-6, IL-17, and G-CSF in each group, detected by ELISA. Values are presented as the mean ± SEM. “***” denotes *p* < 0.001, “**” denotes *p* < 0.01, “*” denotes *p* < 0.05, “ns” denotes *p* > 0.05. Abbreviations used: *P. stercorea*, *Prevotella stercorea*; CIA, collagen-induced arthritis; NT, normal humidity (50%); HT, high humidity (80%); Abx, antibiotics.

To identify microbial features shared or differentially regulated across humidity conditions, Venn diagram analyses were conducted comparing HT-CIA vs. HT-blank mice and NT-CIA vs. NT-blank mice. Four microbial species were commonly altered in both comparisons (Figure 4A). Among these, *Alistipes* sp. and *Alistipes putredinis* were consistently reduced in CIA mice under both humidity conditions relative to their respective controls (Figure 4B). Notably, an unclassified member of the family *Bacteroidaceae* and *P. stercorea* displayed humidity-dependent regulation, being enriched in HT-CIA mice compared with HT-blank controls, but reduced in NT-CIA mice relative to NT-blank controls (Figure 4B).

Given the selective enrichment of *P. stercorea* in CIA mice exposed to high humidity, we next evaluated its potential contribution to arthritis severity in vivo (Figure 4C,D). Oral administration of *P. stercorea* to CIA mice housed under normal humidity resulted in increased arthritis scores (*p* < 0.05), greater ankle swelling, and elevated serum levels of anti-CII IgG (*p* < 0.05), IL-6 (*p* < 0.01), IL-17A (*p* < 0.05), and G-CSF (*p* < 0.01) (Figure 4E–L). In contrast, under high humidity conditions, *P. stercorea* administration exerted more limited effects on arthritis severity, with significant increases observed only in serum IL-17A (*p* < 0.05) and G-CSF (*p* < 0.05) levels (Figure 4K,L and Figure S2C).

The attenuated impact of exogenous *P. stercorea* under high humidity conditions may reflect the already elevated abundance of endogenous *P. stercorea* in CIA mice housed in a high-humidity environment.

### 3.5. Integrated Metagenomic and Metabolomic Analyses Reveal Enhanced Glycosaminoglycan Degradation Under High Humidity

To further elucidate the functional consequences of humidity-associated enrichment of *P. stercorea*, fecal metabolomic profiling was performed in CIA mice under high and normal humidity conditions.

**Figure 5:**
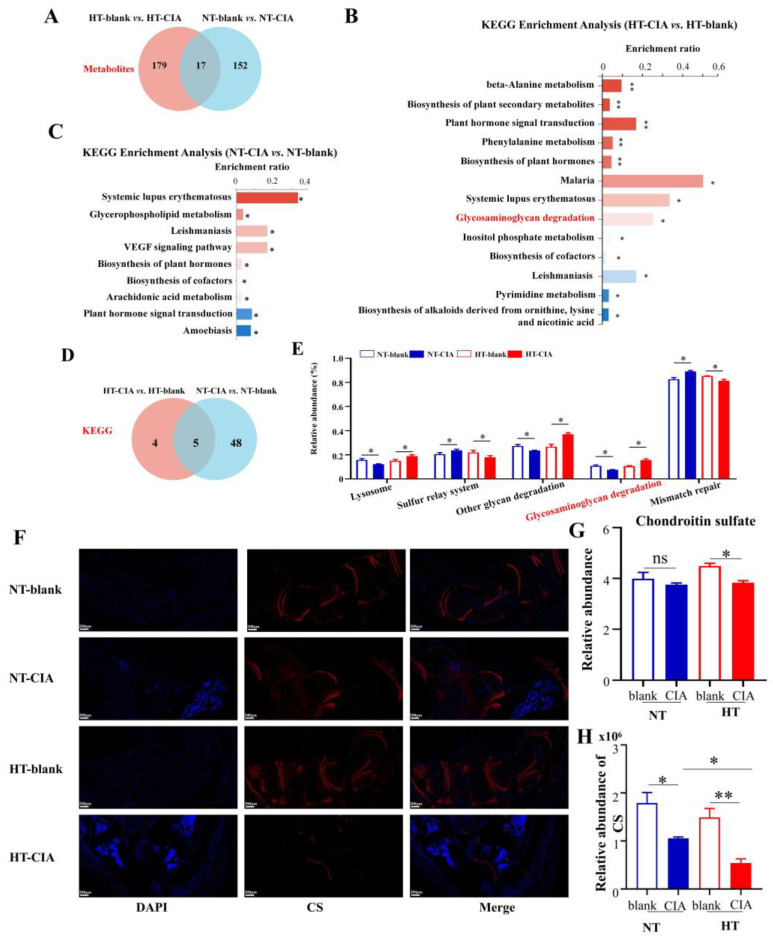
Integrated multi-omics analysis links high humidity to altered glycosaminoglycan metabolism. (**A**) Venn diagram showing the overlap of differential metabolites identified in the NT-CIA vs. NT-blank and HT-CIA vs. HT-blank comparisons. (**B**) KEGG pathway enrichment analysis of differential colonic metabolites between HT-blank and HT-CIA groups. (**C**) KEGG pathway enrichment analysis of differential colonic metabolites between NT-blank and NT-CIA groups. (**D**) Venn diagram showing the overlap of significantly enriched KEGG pathways identified in the NT-CIA vs. NT-blank and HT-CIA vs. HT-blank comparisons. (**E**) Relative abundance of glycosaminoglycan degrading gut microbiota across the NT-blank, NT-CIA, HT-blank, and HT-CIA groups. (**F**) Representative immunofluorescence images of chondroitin sulfate (CS, red) in articular cartilage, with DAPI (blue) nuclear counterstaining. (**G**) Relative abundance of fecal CS identified from the LC-MS/MS-based fecal metabolomics dataset. (**H**) Quantitative analysis of CS immunofluorescence intensity in articular cartilage from each group. Values are presented as the mean ± SEM. “***” denotes *p* < 0.001, “**” denotes *p* < 0.01, “*” denotes *p* < 0.05, “ns” denotes *p* > 0.05. Abbreviations used: CIA, collagen-induced arthritis; NT, normal humidity (50%); HT, high humidity (80%); CS, chondroitin sulfate.

Compared with humidity-matched control mice, CIA mice exhibited substantial alterations in fecal metabolite profiles, with 196 differential metabolites identified under high humidity and 169 under normal humidity conditions (Figure S7). Venn diagram analysis comparing HT-CIA vs. HT-blank and NT-CIA vs. NT-blank groups revealed 17 metabolites that were commonly altered under both humidity conditions (Figure 5A). KEGG pathway enrichment analysis based on differential metabolites identified 13 enriched pathways under high humidity and 9 under normal humidity, among which five pathways were shared between the two conditions (Figure 5B,C). Notably, glycosaminoglycan degradation was enriched under high humidity conditions.

Consistent with the metabolomic findings, metagenomic functional analysis identified five shared KEGG pathways between HT-CIA and NT-CIA comparisons. Among these, lysosome, other glycan degradation, and glycosaminoglycan degradation pathways were enriched in HT-CIA mice compared with HT-blank controls but reduced in NT-CIA mice relative to NT-blank controls (Figure 5D). In contrast, sulfur relay system and mismatch repair pathways displayed the opposite pattern, being enriched under normal humidity but reduced under high humidity conditions (Figure 5E). Together, metagenomic and metabolomic analyses converged on enhanced glycosaminoglycan degradation as a distinguishing functional feature associated with high humidity exposure in CIA mice.

Among metabolites associated with the glycosaminoglycan degradation pathway, a CS-related metabolomic feature was identified in the fecal LC–MS/MS dataset. Its relative abundance did not differ significantly between CIA and control mice under normal humidity, whereas a marked reduction was observed in CIA mice exposed to high humidity (Figure 5G; *p* < 0.05).

To assess whether altered CS metabolism was also reflected at the joint level, immunofluorescence staining was performed to examine CS distribution in articular cartilage. Type II collagen immunization reduced CS distribution under both humidity conditions (Figure 5F–H; *p* < 0.05). Importantly, CIA mice housed under high humidity exhibited a further reduction in CS abundance within joint tissue compared with mice maintained under normal humidity (Figure 5F–H; *p* < 0.05). These findings indicate that high humidity is associated with enhanced glycosaminoglycan degradation signatures and reduced CS abundance in articular cartilage during CIA progression.

### 3.6. Chondroitin Sulfate Supplementation Differentially Modulates Arthritis Severity Under Distinct Humidity Conditions

**Figure 6:**
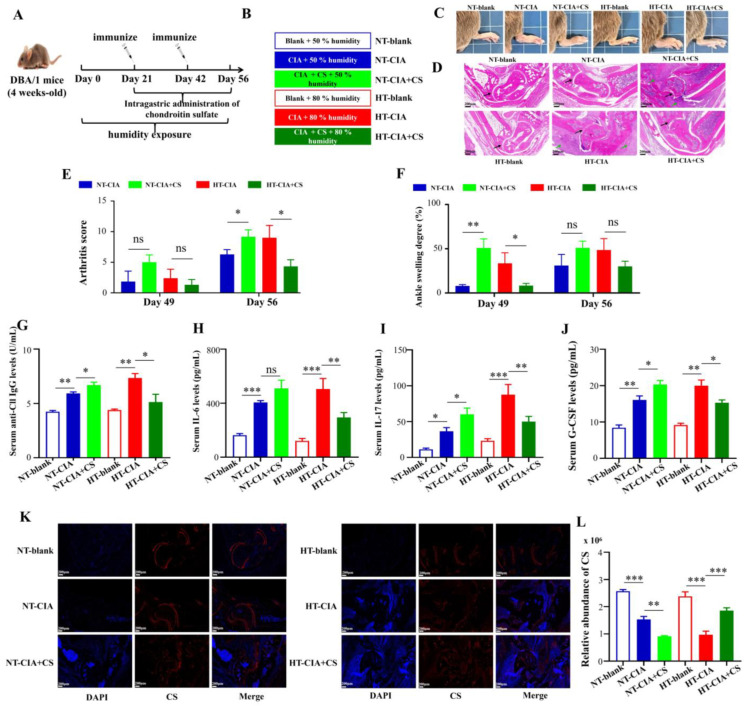
Effect of CS supplementation on arthritis symptoms in CIA mice under different humidity conditions. (**A**) Overview of study design. 4-week-old DBA/1 mice were immunized on Day 21 and Day 42 for CIA modeling and exposed to different humidity conditions from Day 0 to Day 56. CS was administered via intragastric gavage from Day 21 to Day 56. (**B**) Group assignment: NT-blank (blank control with normal humidity, 50%), NT-CIA (CIA model with normal humidity, 50%), NT-CIA+CS (CIA model with 50% humidity receiving CS), HT-blank (blank control with high humidity, 80%), HT-CIA (CIA model with high humidity, 80%), and HT-CIA+CS (CIA model with 80% humidity receiving CS). (**C**) Representative images showing ankle swelling in each group. (**D**) Representative H&E-stained histological sections of ankle joints. Black arrows indicate intact articular cartilage structure, and green arrows indicate inflammatory cell infiltration and synovial hyperplasia in joint tissues (**E**) Arthritis scores in each group on Day 49 and Day 56. (**F**) Ankle swelling degree (%) in each group on Day 49 and Day 56. (**G**–**J**) Serum levels of anti-CII IgG, IL-6, IL-17, and G-CSF in each group, detected by ELISA. (**K**) Representative immunofluorescence images of CS (red) in articular cartilage, with DAPI (blue) nuclear counterstaining. (**L**) Quantitative analysis of CS immunofluorescence intensity in articular cartilage from each group. Values are presented as the mean ± SEM. “***” denotes *p* < 0.001, “**” denotes *p* < 0.01, “*” denotes *p* < 0.05, “ns” denotes *p* > 0.05. Abbreviations used: CS, chondroitin sulfate; CIA, collagen-induced arthritis; NT, normal humidity (50%); HT, high humidity (80%).

To assess the functional relevance of CS in humidity-associated arthritis progression, CIA mice were orally administered CS under normal and high humidity conditions (Figure 6A,B). Under normal humidity conditions, CS supplementation was associated with aggravated arthritis manifestations, including increased inflammatory cell infiltration, earlier disease onset, more severe joint inflammation and swelling, and elevated serum levels of anti-CII IgG, IL-17, and G-CSF (Figure 6C–J and Figure S2D; *p* < 0.05). In contrast, under high humidity conditions, CS supplementation was associated with attenuated arthritis severity, as reflected by reduced arthritis scores, improved joint histopathology, and decreased serum levels of anti-CII IgG, IL-6, IL-17, and G-CSF (Figure 6C–J and Figure S2D; *p* < 0.05).

Consistent with these divergent phenotypic outcomes, immunofluorescence analysis of articular cartilage revealed humidity-dependent differences in CS distribution. Oral CS administration reduced CS abundance in joint tissue of CIA mice under normal humidity, whereas it increased CS distribution in CIA mice exposed to high humidity (Figure 6K,L; *p* < 0.01).

These findings indicate that the effects of CS supplementation on arthritis severity are influenced by environmental humidity.

### 3.7. P. stercorea Exhibits Chondroitin Sulfate-Degrading Capacity

The humidity-dependent effects of CS supplementation prompted further investigation into gut microbial species capable of degrading CS.

**Figure 7:**
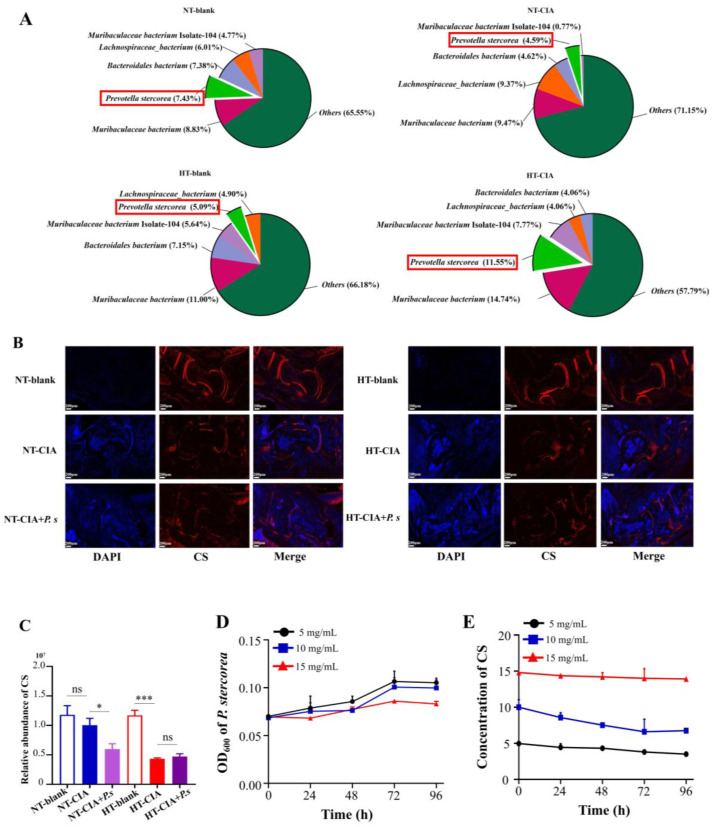
*P. stercorea* exhibits chondroitin sulfate-degrading capacity in CIA mice. (**A**) Pie charts showing the composition of gut microbiota at the species level in NT-blank, NT-CIA, HT-blank, and HT-CIA mice, highlighting the relative abundance of *P. stercorea*. (**B**) Representative immunofluorescence images of CS (red) in articular cartilage, with DAPI (blue) nuclear counterstaining, showing the relative fluorescence intensity of CS in CIA mice supplemented with *P. stercorea*. (**C**) Quantitative analysis of CS relative abundance in articular cartilage from each group. (**D**) Growth curves of *P. stercorea* cultured in vitro under different CS concentrations (5, 10, and 15 mg/mL), measured by OD600 absorbance over 96 h. (**E**) Degradation curves of CS by *P. stercorea* in vitro, showing residual CS concentration over 96 h at different initial CS concentrations. Values are presented as the mean ± SEM. “***” denotes *p* < 0.001, “**” denotes *p* < 0.01, “*” denotes *p* < 0.05, “ns” denotes *p* > 0.05. Abbreviations used: *P. stercorea*, *Prevotella stercorea*; CIA, collagen-induced arthritis; NT, normal humidity (50%); HT, high humidity (80%).

Metagenomic functional analysis revealed that enrichment of the glycosaminoglycan degradation pathway was consistently associated with the presence of *P. stercorea* in CIA mice under both humidity conditions. Further examination of microbial contributors to this pathway showed that the relative contribution of *P. stercorea* closely reflected its overall abundance within the gut microbiota (Figure 7A).

To assess the impact of *P. stercorea* on CS distribution in joint tissue, immunofluorescence staining was performed following *P. stercorea* supplementation. Under normal humidity conditions, administration of *P. stercorea* was associated with reduced CS abundance in articular cartilage of CIA mice (Figure 7B,C; *p* < 0.05), whereas no appreciable change in joint CS distribution was observed under high humidity conditions (Figure 7B,C). In addition, in vitro growth assays demonstrated that *P. stercorea* could utilize CS as a carbon source, accompanied by measurable CS degradation (Figure 7D,E).

Collectively, these results support that *P. stercorea* possesses CS-degrading capacity and may contribute to enhanced glycosaminoglycan degradation observed in CIA mice, particularly under high humidity conditions.

### 3.8. Chondroitin Sulfate Supplementation Attenuates P. stercorea–Associated Arthritis Severity Under High Humidity

**Figure 8:**
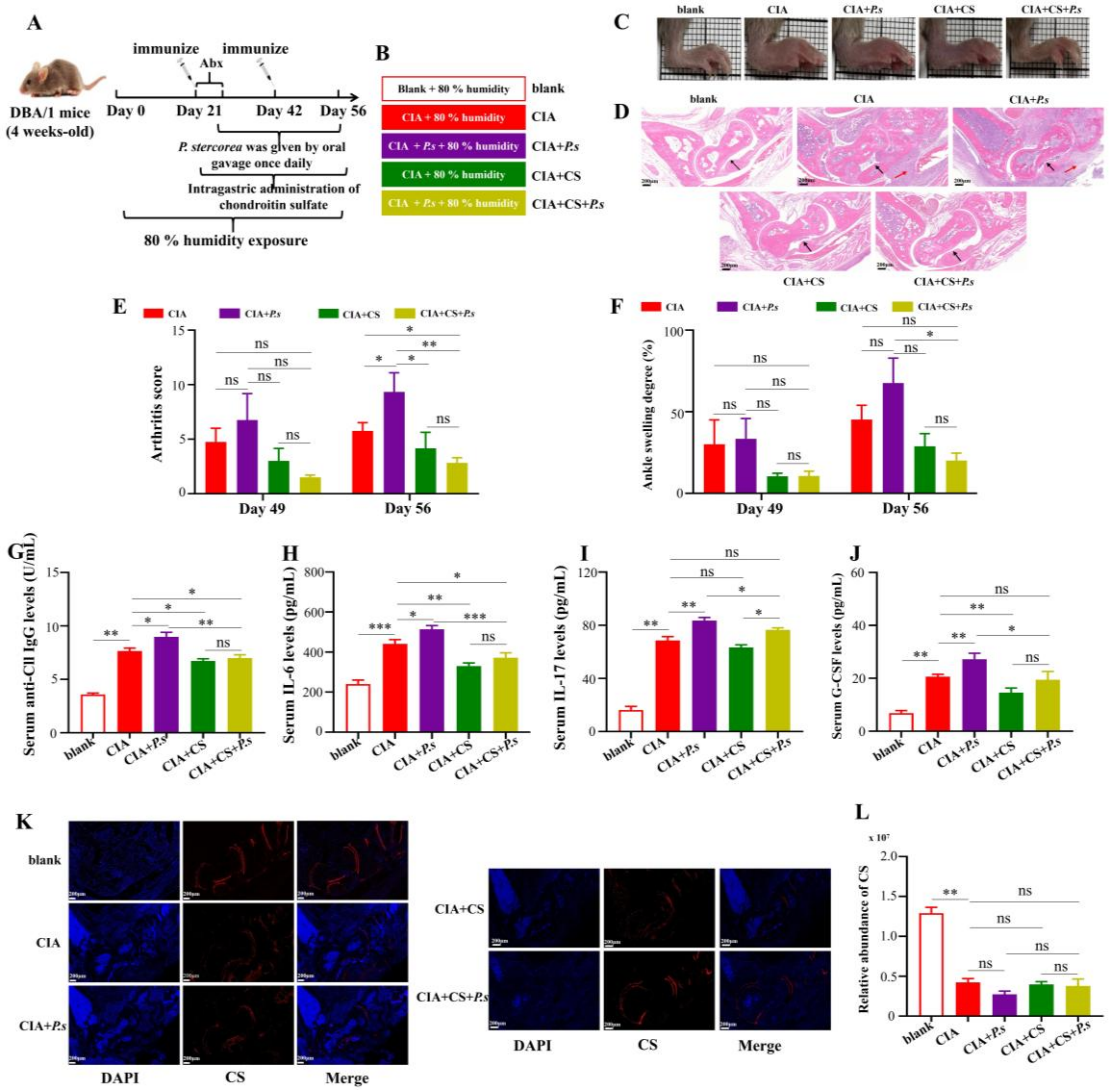
CS supplementation attenuates *P. stercorea*-associated arthritis under high humidity. (**A**) Overview of study design. 4-week-old DBA/1 mice were immunized on Day 21 and Day 42 for CIA modeling. Abx were administered at 200 μL/day from Day 22 to Day 24 and exposed to different humidity conditions from Day 0 to Day 56. *P. stercorea* was administered by oral gavage once daily from Day 25 to Day 56, and CS was administered via intragastric gavage from Day 21 to Day 56. (**B**) Group assignment: blank (control with 80% humidity), CIA (CIA model with high humidity, 80%), CIA+*P. stercorea* (CIA model with 80% humidity receiving *P. stercorea*), CIA+CS (CIA model with 80% humidity receiving CS), and CIA+CS+*P. stercorea* (CIA model with 80% humidity receiving both CS and *P. stercorea*). (**C**) Representative images showing ankle swelling in each group. (**D**) Representative H&E-stained histological sections of ankle joints. Black arrows indicate intact articular cartilage structure, and red arrows indicate inflammatory cell infiltration and synovial hyperplasia in joint tissues (**E**) Arthritis scores in each group on Day 49 and Day 56. (**F**) Ankle swelling degree (%) in each group on Day 49 and Day 56. (**G**–**J**) Serum levels of anti-CII IgG, IL-6, IL-17, and G-CSF in each group, detected by ELISA. (**K**) Representative immunofluorescence images of CS (red) in articular cartilage, with DAPI (blue) nuclear counterstaining. (**L**) Quantitative analysis of CS immunofluorescence intensity in articular cartilage from each group. Values are presented as the mean ± SEM. *** denotes *p* < 0.001, “**” denotes *p* < 0.01, “*” denotes *p* < 0.05, “ns” denotes *p* > 0.05. Abbreviations used: *P. stercorea*, *Prevotella stercorea*; CIA, collagen-induced arthritis; NT, normal humidity (50%); HT, high humidity (80%); Abx, antibiotics.

The effects of *P. stercorea* and CS supplementation on arthritis severity were next evaluated in combination to examine their potential functional interaction under high humidity conditions (Figure 8A,B).

Under high humidity, co-administration of CS and *P. stercorea* was associated with attenuated arthritis severity compared with administration of *P. stercorea* alone. Specifically, CIA mice receiving the combination treatment exhibited improved joint histopathology, lower arthritis scores, reduced joint inflammation and swelling, and decreased representative inflammatory markers, including IL-6 and IL-17A (Figure 8C–J and Figure S2E; *p* < 0.05).

In contrast, except for IL-17A, no significant differences in arthritis-related indices were observed between CIA mice treated with CS alone and those receiving the combined treatment (Figure 8C–J), suggesting that the addition of *P. stercorea* did not further modify the effects of CS supplementation under high humidity.

Immunofluorescence analysis of articular cartilage further supported these observations. CS distribution in joint tissue did not differ significantly between *P. stercorea*-treated mice and mice receiving the combination treatment, or between mice treated with CS alone and those receiving both CS and *P. stercorea* (Figure 8K,L).

These findings are consistent with CS supplementation attenuating the arthritis phenotype associated with *P. stercorea* under high humidity. Because the experiments did not trace gut-derived CS into cartilage or establish a direct transport pathway, the results support an association and functional interaction rather than a fully demonstrated gut-to-cartilage causal sequence.

## 4. Discussion

**Figure 9:**
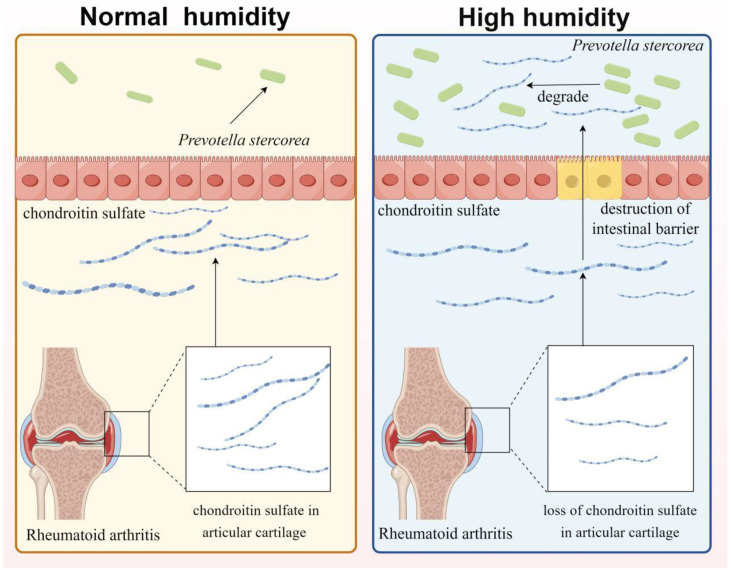
Schematic illustration of the proposed mechanism by which high humidity aggravates RA through *P. stercorea*-associated glycosaminoglycan degradation in CIA mice. Under normal humidity conditions, intestinal barrier integrity is relatively preserved, *P. stercorea* abundance remains low, and CS is maintained at higher levels in the gut and articular cartilage. In contrast, high humidity is associated with impaired intestinal barrier markers and enrichment of *P. stercorea*. The increased CS-degrading capacity of *P. stercorea* may enhance gut microbial glycosaminoglycan degradation, reduce CS availability, and contribute to decreased CS abundance in articular cartilage. These interconnected changes may ultimately amplify systemic inflammation and exacerbate CIA.

The present study identifies a set of interconnected humidity-associated changes in CIA mice. High humidity aggravated joint inflammation, altered gut microbial composition, impaired intestinal barrier markers, enriched microbial glycosaminoglycan degradation functions, and reduced CS abundance in articular cartilage. FMT altered arthritis severity according to donor humidity condition, supporting a microbiota-dependent contribution to humidity-associated disease aggravation. Based on metagenomic screening, *P. stercorea* was prioritized because its abundance and contribution to the glycosaminoglycan degradation pathway were humidity dependent. Its ability to utilize and degrade CS in vitro, together with bacterial supplementation and CS intervention experiments, provides functional support for a microbiota-CS-joint relationship. Collectively, these findings support a humidity-sensitive gut microbiota–cartilage–immune axis in CIA progression (Figure 9).

### 4.1. High Humidity Reshapes the Gut Microenvironment in CIA Mice

The physiological route by which ambient humidity reshapes the gut environment remains incompletely defined. Previous studies have suggested that high humidity or humid heat exposure may influence heat dissipation, fluid balance, stress-related signaling, intestinal epithelial homeostasis, and gut microbial ecology [36,37,38,39,40,41,42]. These processes provide a general physiological context for understanding why the gut barrier and microbiota may be sensitive to humidity-related environmental changes. In the present study, our direct observations showed that high humidity was associated with altered gut microbial profiles, colonic histological changes, and reduced ZO-1 and occludin staining in CIA mice. Therefore, further studies are required to define the upstream host pathways that connect environmental humidity to intestinal barrier impairment and microbial remodeling.

Clinical observations have long suggested an association between humid environments and worsened arthritis symptoms [43]; however, the biological basis of this relationship has remained unclear. Previous studies have mainly described epidemiological or symptomatic associations, leaving the underlying mechanisms insufficiently explored [44,45]. In the present study, high humidity not only aggravated arthritis severity but also induced consistent alterations in gut microbial composition and microbial functional profiles. The FMT experiments further demonstrated that transferring microbiota between humidity conditions modulates arthritis severity, supporting a microbiota-dependent contribution to humidity-associated inflammation. These findings provide functional evidence that gut microbiota contributes to humidity-associated arthritis exacerbation and suggest that environmental humidity may influence systemic inflammatory responses, at least in part, through remodeling of the gut microbial ecosystem.

Reduced ZO-1 and occludin staining, together with increased colonic inflammatory features, is consistent with impaired intestinal barrier integrity in high-humidity CIA mice. Intestinal barrier dysfunction can facilitate microbial antigen exposure and systemic inflammatory signaling [36,39,40], providing a biologically plausible link to the increased IL-6 and IL-17A observed in this study. In this context, the FMT results further support the functional involvement of gut microbiota in humidity-associated inflammatory amplification. However, microbial translocation and the host signaling pathways connecting barrier impairment to joint inflammation were not directly examined in the present study and should be addressed in future work.

### 4.2. Humidity-Associated Functional Remodeling of Gut Microbiota

*P. stercorea* was prioritized as a candidate functional taxon based on the metagenomic findings in the present study. This species was enriched in HT-CIA mice relative to HT-blank controls, showed an opposite trend under normal humidity, and contributed prominently to the glycosaminoglycan degradation pathway. Thus, its selection was supported by the convergence of taxonomic abundance, pathway contribution, and humidity-specific regulation. This data-driven prioritization is important because associations between *Prevotella* species and arthritis have been reported to vary across studies [46,47,48]. Our findings suggest that, in the context of humidity-associated arthritis exacerbation, the functional metabolic capacity of a microbial species may be more informative than taxonomic abundance alone.

Integrated metagenomic and fecal metabolomic analyses converged on enrichment of glycosaminoglycan degradation under high humidity, indicating that humidity exposure was associated not only with changes in microbial composition but also with functional metabolic remodeling.

### 4.3. Microbial Glycosaminoglycan Metabolism and Cartilage CS Alteration

Glycosaminoglycans are essential components of the cartilage extracellular matrix [49,50], and inflammatory, infectious, or mechanical stress can accelerate their release or degradation [51,52,53]. In the present study, CS abundance in articular cartilage was lowest in high-humidity CIA mice, while *P. stercorea* showed CS-degrading capacity in vitro. These findings connect the humidity-associated enrichment of microbial glycosaminoglycan degradation functions with altered cartilage CS homeostasis. Nevertheless, fecal metabolite profiling and cartilage immunofluorescence reflect different biological compartments. Therefore, without isotope tracing or another direct transport experiment, intestinal CS degradation cannot be claimed to cause cartilage CS depletion. Rather, the present data support a testable model in which high humidity promotes microbial functional remodeling and inflammatory amplification, which may jointly contribute to impaired CS homeostasis during arthritis progression.

### 4.4. Humidity-Dependent Effects of CS Supplementation

The divergent effects of oral CS under the two humidity conditions suggest that its impact on arthritis may depend on the environmental and microbial context. Under high humidity, where microbial glycosaminoglycan degradation was enriched and tissue CS was reduced, CS supplementation was associated with improved arthritis outcomes. In contrast, under normal humidity, CS supplementation was associated with worsened arthritis manifestations and reduced cartilage CS staining. The mechanism underlying this humidity-dependent divergence remains unclear. One possible explanation is that high humidity reshapes the gut microbial ecosystem in a way that alters CS utilization, degradation-product formation, intestinal absorption, or downstream inflammatory responses. However, these possibilities were not directly tested in the present study. Therefore, our findings should not be interpreted as evidence that CS supplementation is uniformly protective in arthritis. Rather, they suggest that the biological effects of oral CS may be context dependent and influenced by humidity-associated microbial metabolism. Further studies are required to determine whether differences in microbial CS utilization, degradation-product formation, intestinal absorption, or immune responses account for the divergent effects observed under normal and high humidity conditions.

### 4.5. Limitations

Several limitations of this study should be acknowledged. First, our findings were obtained from a murine CIA model, and further studies are required to determine whether similar humidity-sensitive microbiota–cartilage interactions occur in human RA. Second, although integrated multi-omics analysis, *P. stercorea* supplementation, in vitro CS degradation, and CS intervention experiments support a microbiota–CS–joint relationship, the evidence directly linking intestinal CS degradation to articular cartilage CS depletion remains indirect. Fecal CS levels and cartilage CS abundance were measured in different biological compartments, and isotope-tracing or direct transport experiments were not performed. In addition, the oral bioavailability of CS is relatively limited and variable; therefore, the effects of oral CS supplementation should not be interpreted as direct evidence of systemic replacement of cartilage CS. Rather, these effects may reflect combined interactions among orally administered CS, gut microbial metabolism, intestinal processing, and downstream changes in cartilage CS abundance and arthritis severity. Therefore, the proposed gut microbiota–CS–cartilage axis should be interpreted as a supported mechanistic model rather than a fully resolved linear causal pathway. Third, although *P. stercorea* emerged as a humidity-associated functional candidate, additional microbial species and metabolites may also participate in glycosaminoglycan metabolism under high-humidity conditions. Fourth, the upstream host pathways connecting environmental humidity to intestinal barrier impairment and microbial remodeling, as well as the immune pathways linking cartilage metabolic disruption to inflammatory amplification, were not fully defined. Finally, the mechanism underlying the humidity-dependent effects of CS supplementation remains unclear and warrants further investigation. Despite these limitations, the present findings provide experimental evidence linking high humidity to microbiota-dependent metabolic remodeling and arthritis exacerbation. Understanding how climate-related environmental exposures influence microbial function and host tissue metabolism may help refine preventive or adjunctive strategies for immune-mediated inflammatory diseases.

## 5. Conclusions

In summary, high humidity aggravated CIA and was associated with microbiota-dependent remodeling, impaired intestinal barrier markers, enrichment of glycosaminoglycan degradation functions, and reduced cartilage CS. *P. stercorea* emerged from the present multi-omics dataset as a humidity-associated functional candidate and demonstrated CS-degrading capacity. The intervention results support a context-dependent interaction among humidity, *P. stercorea*, CS metabolism, and arthritis severity. Direct tracing and host mechanism studies are now needed before the proposed gut microbiota–cartilage axis can be considered fully causal or translated into humidity-control or microbiome-targeted clinical recommendations.

## Data Availability

The raw sequences of metagenomic sequencing from DBA/1 mice have been submitted to the NCBI Sequence Read Archive under accession numbers SRR22941739-SRR22941782. Further information and requests for resources and reagents should be directed to the corresponding author, C.W.

## Supplementary Materials

The following supporting information can be downloaded at: https://www.mdpi.com/article/10.3390/microorganisms14071540/s1, Figure S1: Humidity (A) and temperature (B) variation in the man-made climate box; Figure S2: Line charts depicting the dynamic changes in arthritis scores across five independent murine experiments; Figure S3: LEfSe identified the differential microbial species between HT-CIA and HT-blank. Significant differences are shown (LDA score > 2). HT-CIA: the CIA mice under high humidity; HT-blank: the control mice under high humidity; Figure S4: LEfSe identified the differential KEGG pathways between HT-CIA and HT-blank. Significant differences are shown (LDA score > 2). HT-CIA: the CIA mice under high humidity; HT-blank: the control mice under high humidity; Figure S5: LEfSe identified the differential microbial species between NT-CIA and NT-blank. Significant differences are shown (LDA score > 2). NT-CIA: the CIA mice under normal humidity; NT-blank: the control mice under normal humidity; Figure S6: LEfSe identified the differential KEGG pathways between NT-CIA and NT-blank. Significant differences are shown (LDA score > 2). NT-CIA: the CIA mice under normal humidity; NT-blank: the control mice under normal humidity; Figure S7: Volcano plot of different fecal metabolites in NT-blank (A) vs. NT-CIA and HT-blank vs. HT-CIA (B); Table S1: Dietary composition of the standard laboratory diet used in this study; Table S2: Statistical tests, correction methods, and exact *p* values for the major prespecified comparisons.

## Abbreviations

The following abbreviations are used in this manuscript:

RA: rheumatoid arthritis; CIA: collagen-induced arthritis; CS: chondroitin sulfate; *P. stercorea*: *Prevotella stercorea*; FMT: fecal microbiota transplantation; anti-CII IgG: anti-collagen type II antibody; IL-6: interleukin-6; IL-17A: interleukin-17A; G-CSF: granulocyte colony stimulating factor; H&E: hematoxylin and eosin staining; LC-MS/MS: liquid chromatography-tandem mass spectrometry; NT: normal humidity; HT: high humidity; KEGG: kyoto encyclopedia of genes and genomes; Abx: antibiotics.

## References

[1] Weyand C.M., Goronzy J.J. (2021). The immunology of rheumatoid arthritis. Nat. Immunol..

[2] Black R.J., Cross M., Haile L.M., Culbreth G.T., Steinmetz J.D., Hagins H., Kopec J.A., Brooks P.M., Woolf A.D., Ong K.L. (2023). Global, regional, and national burden of rheumatoid arthritis, 1990–2020, and projections to 2050: A systematic analysis of the Global Burden of Disease Study 2021. Lancet Rheumatol..

[3] Björk M., Dragioti E., Alexandersson H., Esbensen B.A., Boström C., Friden C., Hjalmarsson S., Hörnberg K., Kjeken I., Regardt M. (2022). Inflammatory arthritis and the effect of physical activity on quality of life and self-reported function: A systematic review and meta-analysis. Arthritis Care Res..

[4] Scherer H.U., Häupl T., Burmester G.R. (2020). The etiology of rheumatoid arthritis. J. Autoimmun..

[5] Zaiss M.M., Joyce Wu H.-J., Mauro D., Schett G., Ciccia F. (2021). The gut–joint axis in rheumatoid arthritis. Nat. Rev. Rheumatol..

[6] Wang M., Chen J., Lin X., Huang L., Li H., Wen C., He Z. (2021). High humidity aggravates the severity of arthritis in collagen-induced arthritis mice by upregulating xylitol and L-pyroglutamic acid. Arthritis Res. Ther..

[7] Kudo E., Song E., Yockey L.J., Rakib T., Wong P.W., Homer R.J., Iwasaki A. (2019). Low ambient humidity impairs barrier function and innate resistance against influenza infection. Proc. Natl. Acad. Sci. USA.

[8] Davis R.E., McGregor G.R., Enfield K.B. (2016). Humidity: A review and primer on atmospheric moisture and human health. Environ. Res..

[9] Myllykangas-Luosujarvi R., Seuri M., Husman T., Korhonen R., Pakkala K., Aho K. (2002). A cluster of inflammatory rheumatic diseases in a moisture-damaged office. Clin. Exp. Rheumatol..

[10] Patberg W.R., Rasker J.J. (2004). Weather effects in rheumatoid arthritis: From controversy to consensus. A review. J. Rheumatol..

[11] Mori H., Sawada T., Nishiyama S., Shimada K., Tahara K., Hayashi H., Kato E., Tago M., Matsui T., Tohma S. (2019). Influence of seasonal changes on disease activity and distribution of affected joints in rheumatoid arthritis. BMC Musculoskelet. Disord..

[12] Pickard J.M., Zeng M.Y., Caruso R., Núñez G. (2017). Gut microbiota: Role in pathogen colonization, immune responses, and inflammatory disease. Immunol. Rev..

[13] Wells P.M., Adebayo A.S., Bowyer R.C., Freidin M.B., Finckh A., Strowig T., Lesker T.R., Alpizar-Rodriguez D., Gilbert B., Kirkham B. (2020). Associations between gut microbiota and genetic risk for rheumatoid arthritis in the absence of disease: A cross-sectional study. Lancet Rheumatol..

[14] Zhang X., Zhang D., Jia H., Feng Q., Wang D., Liang D., Wu X., Li J., Tang L., Li Y. (2015). The oral and gut microbiomes are perturbed in rheumatoid arthritis and partly normalized after treatment. Nat. Med..

[15] Wang B., He Y., Tang J., Ou Q., Lin J. (2020). Alteration of the gut microbiota in tumor necrosis factor-α antagonist-treated collagen-induced arthritis mice. Int. J. Rheum. Dis..

[16] Block K.E., Zheng Z., Dent A.L., Kee B.L., Huang H. (2016). Gut microbiota regulates K/BxN autoimmune arthritis through follicular helper T but not Th17 cells. J. Immunol..

[17] Maeda Y., Takeda K. (2019). Host–microbiota interactions in rheumatoid arthritis. Exp. Mol. Med..

[18] Jeong Y., Kim J.-W., You H.J., Park S.-J., Lee J., Ju J.H., Park M.S., Jin H., Cho M.-L., Kwon B. (2019). Gut microbial composition and function are altered in patients with early rheumatoid arthritis. J. Clin. Med..

[19] Jubair W.K., Hendrickson J.D., Severs E.L., Schulz H.M., Adhikari S., Ir D., Pagan J.D., Anthony R.M., Robertson C.E., Frank D.N. (2018). Modulation of inflammatory arthritis in mice by gut microbiota through mucosal inflammation and autoantibody generation. Arthritis Rheumatol..

[20] Aswinanand B., Haridevamuthu B., Guru A., Arockiaraj J. (2025). The impact of climate, weather, seasonal transitions, and diurnal rhythms on gut microbiota and immune homeostasis. Antonie Leeuwenhoek.

[21] Chen S., Zheng Y., Zhou Y., Guo W., Tang Q., Rong G., Hu W., Tang J., Luo H. (2019). Gut Dysbiosis with Minimal Enteritis Induced by High Temperature and Humidity. Sci. Rep..

[22] Deng R., Ma P., Li B., Wu Y., Yang X. (2022). Development of allergic asthma and changes of intestinal microbiota in mice under high humidity and/or carbon black nanoparticles. Ecotoxicol. Environ. Saf..

[23] Wang D., Zheng Z., Yu H., Dou D., Gao Y., Xu S., Li Z., Sun L., Qiu X., Zhong X. (2023). Impact of humid climate on rheumatoid arthritis faecal microbiome and metabolites. Sci. Rep..

[24] Yin H., Zhong Y., Wang H., Hu J., Xia S., Xiao Y., Nie S., Xie M. (2022). Short-term exposure to high relative humidity increases blood urea and influences colonic urea-nitrogen metabolism by altering the gut microbiota. J. Adv. Res..

[25] Obernier J.A., Baldwin R.L. (2006). Establishing an appropriate period of acclimatization following transportation of laboratory animals. ILAR J..

[26] Hayashi H., Shibata K., Sakamoto M., Tomita S., Benno Y. (2007). *Prevotella copri* sp. nov. and *Prevotella stercorea* sp. nov., isolated from human faeces. Int. J. Syst. Evol. Microbiol..

[27] Engebretsen K.A., Johansen J.D., Kezic S., Linneberg A., Thyssen J.P. (2016). The effect of environmental humidity and temperature on skin barrier function and dermatitis. J. Eur. Acad. Dermatol. Venereol..

[28] Simmering J.E., Polgreen L.A., Hornick D.B., Sewell D.K., Polgreen P.M. (2017). Weather-Dependent Risk for Legionnaires’ Disease, United States. Emerg. Infect. Dis..

[29] Rose R., Gilbert H., Loyau T., Giorgi M., Billon Y., Riquet J., Renaudeau D., Gourdine J.L. (2017). Interactions between sire family and production environment (temperate vs. tropical) on performance and thermoregulation responses in growing pigs. J. Anim. Sci..

[30] Kennedy E.A., King K.Y., Baldridge M.T. (2018). Mouse Microbiota Models: Comparing Germ-Free Mice and Antibiotics Treatment as Tools for Modifying Gut Bacteria. Front. Physiol..

[31] Jiang L., Shang M., Yu S., Liu Y., Zhang H., Zhou Y., Wang M., Wang T., Li H., Liu Z. (2022). A high-fiber diet synergizes with *Prevotella copri* and exacerbates rheumatoid arthritis. Cell. Mol. Immunol..

[32] Scher J.U., Sczesnak A., Longman R.S., Segata N., Ubeda C., Bielski C., Rostron T., Cerundolo V., Pamer E.G., Abramson S.B. (2013). Expansion of intestinal *Prevotella copri* correlates with enhanced susceptibility to arthritis. eLife.

[33] Omata T., Itokazu Y., Inoue N., Segawa Y. (2000). Effects of chondroitin sulfate-C on articular cartilage destruction in murine collagen-induced arthritis. Arzneimittelforschung.

[34] Zhou W., Qi D., Swaisgood R.R., Wang L., Jin Y., Wu Q., Wei F., Nie Y. (2021). Symbiotic bacteria mediate volatile chemical signal synthesis in a large solitary mammal species. ISME J..

[35] He Z., Chen H., Chen Y., Sun X., Qiu F., Qiu Y., Wen C., Mao Y., Ye D. (2024). Selenium deficiency induces irritable bowel syndrome: Analysis of UK Biobank data and experimental studies in mice. Ecotoxicol. Environ. Saf..

[36] Dokladny K., Zuhl M.N., Moseley P.L. (2016). Intestinal epithelial barrier function and tight junction proteins with heat and exercise. J. Appl. Physiol..

[37] Lyte M., Vulchanova L., Brown D.R. (2011). Stress at the intestinal surface: Catecholamines and mucosa-bacteria interactions. Cell Tissue Res..

[38] Neuman H., Debelius J.W., Knight R., Koren O. (2015). Microbial endocrinology: The interplay between the microbiota and the endocrine system. FEMS Microbiol. Rev..

[39] Odenwald M.A., Turner J.R. (2017). The intestinal epithelial barrier: A therapeutic target?. Nat. Rev. Gastroenterol. Hepatol..

[40] Suzuki T. (2013). Regulation of intestinal epithelial permeability by tight junctions. Cell. Mol. Life Sci..

[41] Wang F., Lu C., Lei Y., Lei T.H. (2025). Prolonged Humid Heat Triggers Systemic Inflammation and Stress Signaling: Fluid Intake Modulates NF-kappaB, p38, JNK2, and STAT3alpha Pathways. Int. J. Mol. Sci..

[42] Weng H., Deng L., Wang T., Xu H., Wu J., Zhou Q., Yu L., Chen B., Huang L., Qu Y. (2024). Humid heat environment causes anxiety-like disorder via impairing gut microbiota and bile acid metabolism in mice. Nat. Commun..

[43] Guedj D., Weinberger A. (1990). Effect of weather conditions on rheumatic patients. Ann. Rheum. Dis..

[44] Azzouzi H., Ichchou L. (2020). Seasonal and Weather Effects on Rheumatoid Arthritis: Myth or Reality?. Pain Res. Manag..

[45] Hu C., Wu J., Luo Y., Zhu Y., Chang R., Qian S., Ding X. (2026). Association between weather conditions and rheumatoid arthritis: A systematic review and meta-analysis. Autoimmun. Rev..

[46] Alpizar-Rodriguez D., Lesker T.R., Gronow A., Gilbert B., Raemy E., Lamacchia C., Gabay C., Finckh A., Strowig T. (2019). *Prevotella copri* in individuals at risk for rheumatoid arthritis. Ann. Rheum. Dis..

[47] Seifert J.A., Bemis E.A., Ramsden K., Lowell C., Polinski K., Feser M., Fleischer C., Demoruelle M.K., Buckner J., Gregersen P.K. (2023). Association of Antibodies to *Prevotella copri* in Anti-Cyclic Citrullinated Peptide-Positive Individuals At Risk of Developing Rheumatoid Arthritis and in Patients with Early or Established Rheumatoid Arthritis. Arthritis Rheumatol..

[48] Stoll M.L. (2020). Genetics, Prevotella, and the pathogenesis of rheumatoid arthritis. Lancet Rheumatol..

[49] Chen Y., Mehmood K., Chang Y.F., Tang Z., Li Y., Zhang H. (2023). The molecular mechanisms of glycosaminoglycan biosynthesis regulating chondrogenesis and endochondral ossification. Life Sci..

[50] Heath S., Han Y., Hua R., Roy A., Jiang J., Nyman J.S., Wang X. (2023). Assessment of glycosaminoglycan content in bone using Raman spectroscopy. Bone.

[51] Jinno A., Park P.W. (2015). Role of glycosaminoglycans in infectious disease. Methods Mol. Biol..

[52] Szeremeta A., Jura-Poltorak A., Kozma E.M., Glowacki A., Kucharz E.J., Kopec-Medrek M., Olczyk K. (2018). Effects of a 15-month anti-TNF-alpha treatment on plasma levels of glycosaminoglycans in women with rheumatoid arthritis. Arthritis Res. Ther..

[53] Vasan N. (1983). Effects of physical stress on the synthesis and degradation of cartilage matrix. Connect. Tissue Res..

