# Supplementary material for: High Humidity Exacerbates Rheumatoid Arthritis in Mice via Prevotella stercorea-Mediated Chondroitin Sulfate Degradation
Source: Microorganisms. 2026 Jul 14;14(7):1540. doi: 10.3390/microorganisms14071540 (PMC13413999; doi:10.3390/microorganisms14071540)
Supplement: Supplementary file 1 [file microorganisms-14-01540-s001.zip › microorganisms-4329602-supplementary.pdf]

Supplementary Materials

High humidity exacerbates rheumatoid arthritis in mice via *Prevotella stercorea*-mediated chondroitin sulfate degradation

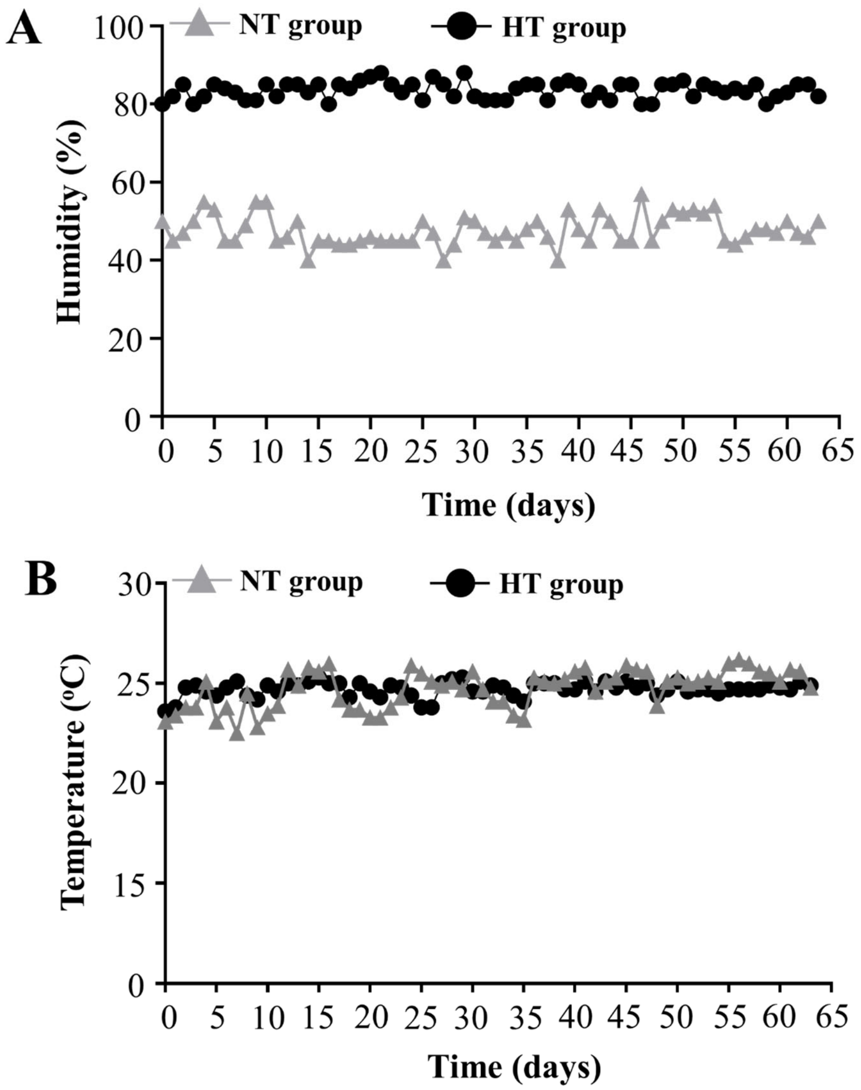

Figure S1. Humidity (A) and temperature (B) variation in the man-made climate box.

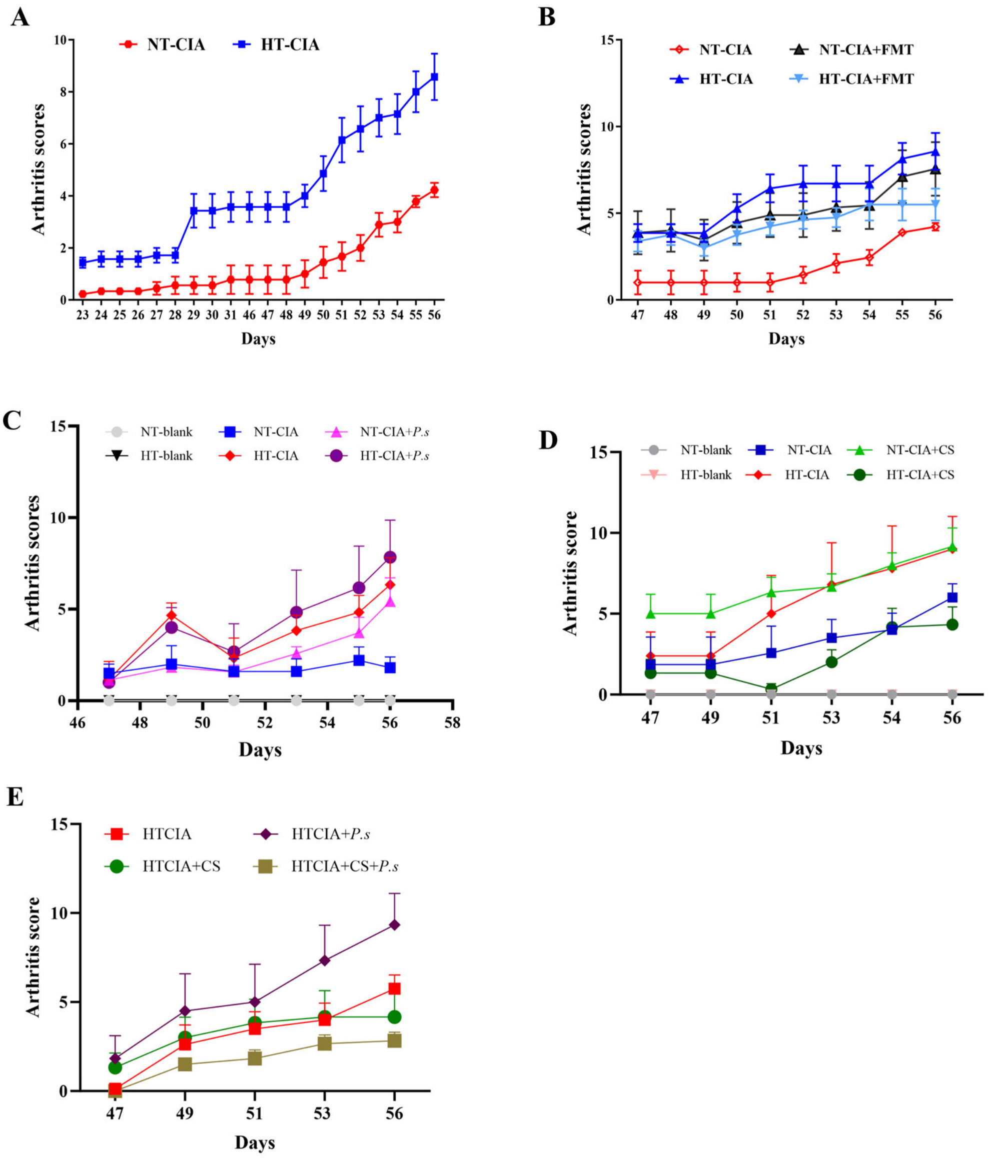

Figure S2. Line charts depicting the dynamic changes in arthritis scores across five independent murine experiments. (A) Arthritis scores of mice in Experiment I; (B) Arthritis scores of mice in Experiment II; (C) Arthritis scores of mice in Experiment III; (D) Arthritis scores of mice in Experiment IV; (E) Arthritis scores of mice in Experiment V.

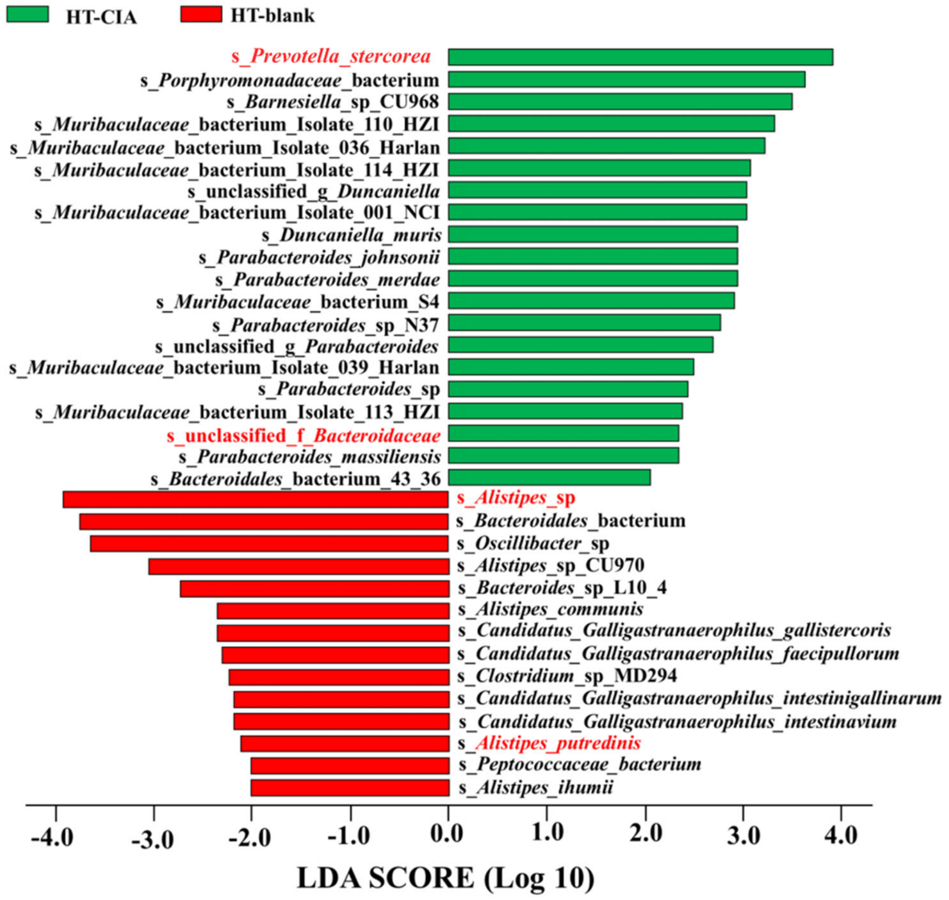

Figure S3. LEfSe identified the differential microbial species between HT-CIA and HT-blank. Significant differences are shown (LDA score >2). HT-CIA: the CIA mice under high humidity; HT-blank: the control mice under high humidity.

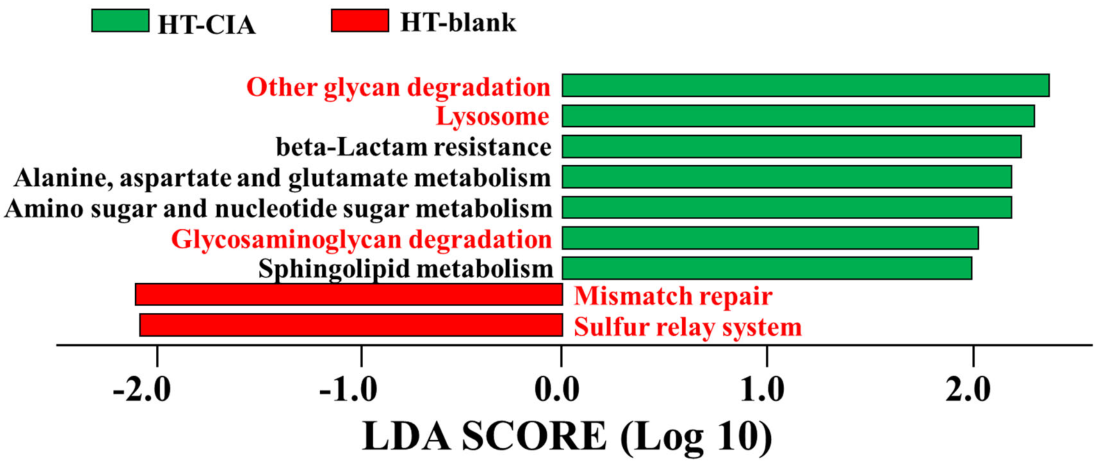

Figure S4. LEfSe identified the differential KEGG pathways between HT-CIA and HT-blank. Significant differences are shown (LDA score >2). HT-CIA: the CIA mice under high humidity; HT-blank: the control mice under high humidity.

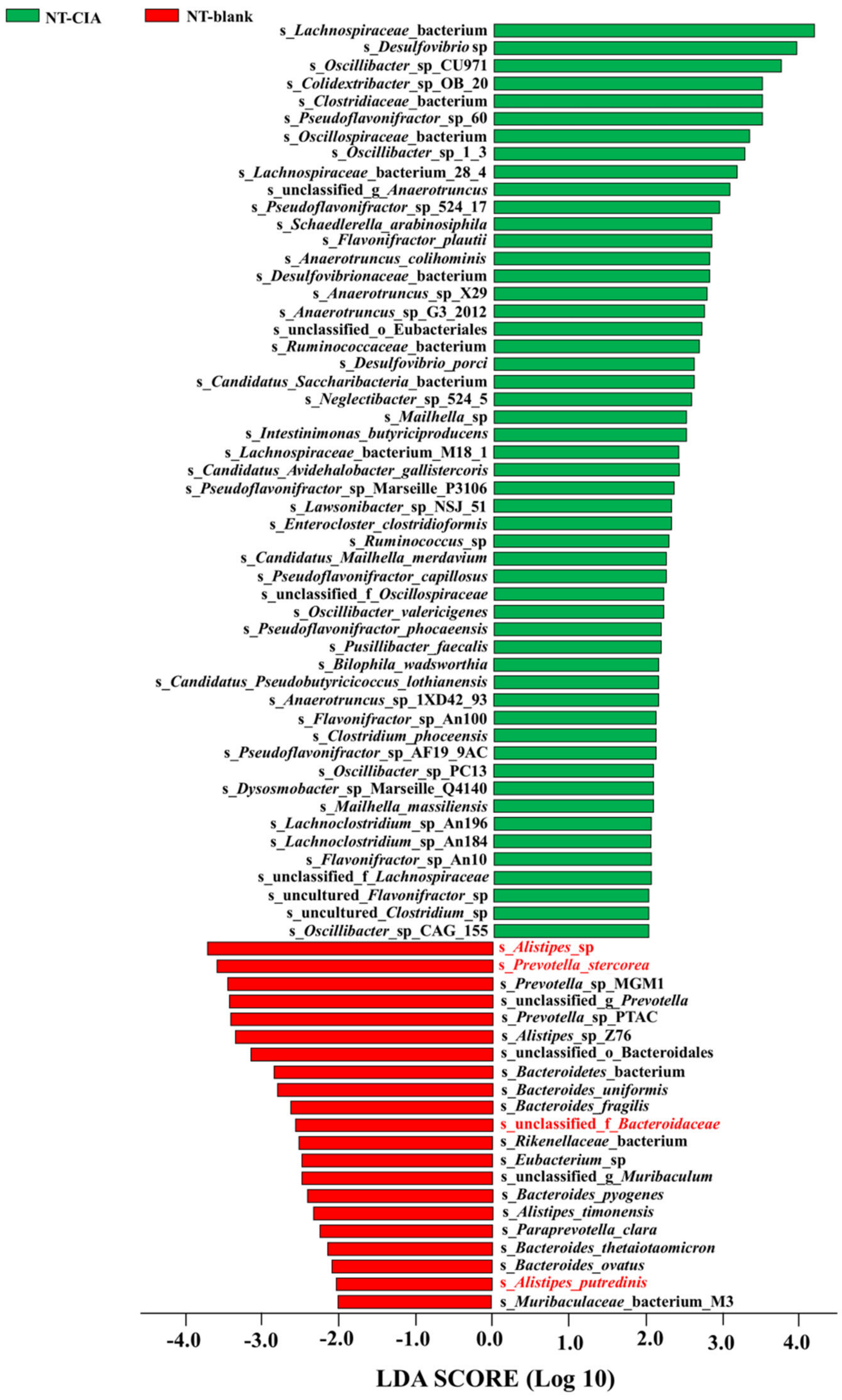

Figure S5. LEfSe identified the differential microbial species between NT-CIA and NT-blank. Significant differences are shown (LDA score >2). NT-CIA: the CIA mice under normal humidity; NT-blank: the control mice under normal humidity.

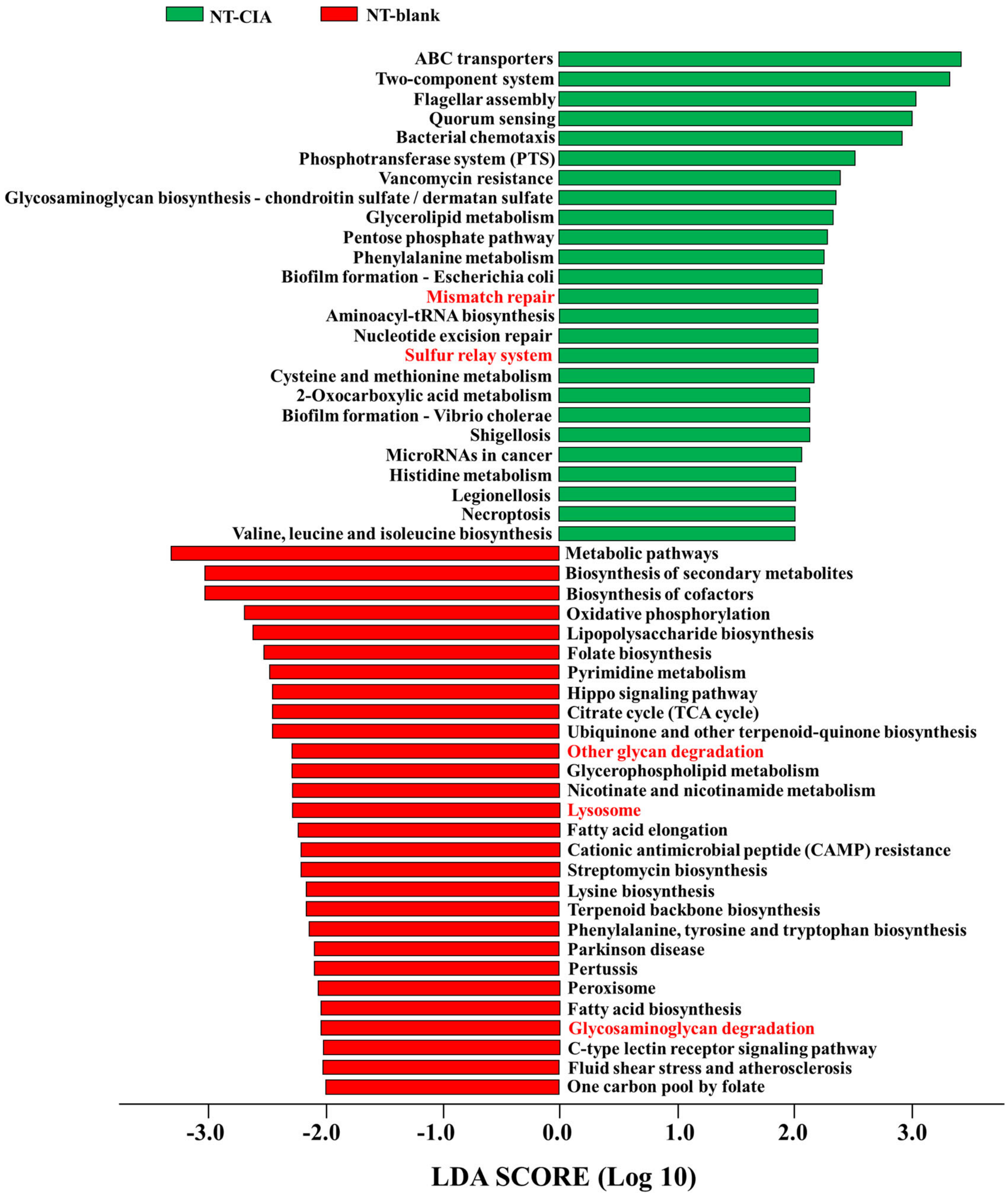

Figure S6. LEfSe identified the differential KEGG pathways between NT-CIA and NT-blank. Significant differences are shown (LDA score >2). NT-CIA: the CIA mice under normal humidity; NT-blank: the control mice under normal humidity.

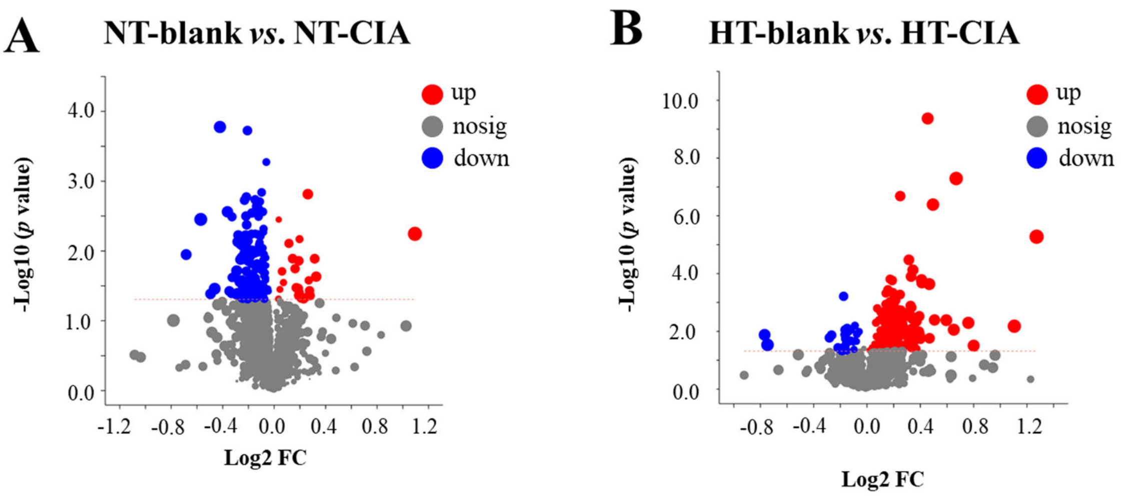

Figure S7. volcano plot of different fecal metabolites in NT-blank (A) *vs*. NT-CIA and HT-blank *vs*. HT-CIA (B).

Table S1. Dietary composition of the standard laboratory diet used in this study.

Category	Composition
Diet name	Irradiated laboratory mouse growth and reproduction diet (CRO)
Manufacturer	Jiangsu Xietong Pharmaceutical Bioengineering Co., Ltd., China
Catalogue number	XTI01CR-010
Main ingredients	Corn, extruded corn, wheat bran, imported milk powder, soybean meal, soybean oil, sodium chloride, limestone powder, dicalcium phosphate, choline chloride, methionine, vitamins, and trace minerals
Moisture	≤100 g/kg
Crude protein	≥200 g/kg
Crude fat	≥40 g/kg
Crude fiber	≤50 g/kg
Crude ash	≤80 g/kg
Calcium	10–18 g/kg
Total phosphorus	6–12 g/kg
Calcium:phosphorus ratio	1.2:1–1.7:1
Gross energy	3902 kcal/kg
Physiological energy	3490 kcal/kg
Energy contribution from protein	24.0%
Energy contribution from fat	15.0%
Energy contribution from carbohydrate	61.0%

Table S2. Statistical tests, correction methods, and exact *p* values for the major prespecified comparisons.

Figure panel	Outcome/parameter	Comparison	Statistical test	Correction method	Raw *p* value	Adjusted *p* value	Significance after correction
Fig. 1E	Arthritis score, Day 56	HT-CIA vs NT-CIA	Two-way ANOVA	Holm–Šídák multiple-comparison correction	<0.0010	0.0060	***
Fig. 1H	Serum IL-17A	HT-CIA vs NT-CIA	One-way ANOVA	Holm–Šídák multiple-comparison correction	0.0100	0.0100	*
Fig. 1I	Serum IL-6	HT-CIA vs NT-CIA	One-way ANOVA	Holm–Šídák multiple-comparison correction	0.0400	0.0100	*
Fig. 3F	Arthritis score	NT-CIA+FMT vs NT-CIA	One-way ANOVA	Holm–Šídák multiple-comparison correction	0.0030	0.0060	**
Fig. 3F	Arthritis score	HT-CIA+FMT vs HT-CIA	One-way ANOVA	Holm–Šídák multiple-comparison correction	0.0400	0.0400	*
Fig. 3I	Serum IL-6	NT-CIA+FMT vs NT-CIA	One-way ANOVA	Holm–Šídák multiple-comparison correction	0.0200	0.0200	*
Fig. 3I	Serum IL-6	HT-CIA+FMT vs HT-CIA	One-way ANOVA	Holm–Šídák multiple-comparison correction	0.0020	0.0040	**
Fig. 3J	Serum IL-17A	NT-CIA+FMT vs NT-CIA	One-way ANOVA	Holm–Šídák multiple-comparison correction	0.4400	0.4400	ns
Fig. 3J	Serum IL-17A	HT-CIA+FMT vs HT-CIA	One-way ANOVA	Holm–Šídák multiple-comparison correction	<0.0010	<0.001	***
Fig. 4G	Arthritis score, Day 56	NT-CIA+*P. stercorea* vs NT-CIA	Two-way ANOVA	Holm–Šídák multiple-comparison correction	0.0200	0.0400	*
Fig. 4G	Arthritis score, Day 56	HT-CIA+*P. stercorea* vs HT-CIA	Two-way ANOVA	Holm–Šídák multiple-comparison correction	0.4600	0.4600	ns
Fig. 4J	Serum IL-6	NT-CIA+*P. stercorea* vs NT-CIA	One-way ANOVA	Holm–Šídák multiple-comparison correction	0.0007	0.0027	**
Fig. 4J	Serum IL-6	HT-CIA+*P. stercorea* vs HT-CIA	One-way ANOVA	Holm–Šídák multiple-comparison correction	0.9239	0.9239	ns
Fig. 4K	Serum IL-17A	NT-CIA+*P. stercorea* vs NT-CIA	One-way ANOVA	Holm–Šídák multiple-comparison correction	0.0149	0.0296	*
Fig. 4K	Serum IL-17A	HT-CIA+*P. stercorea* vs HT-CIA	One-way ANOVA	Holm–Šídák multiple-comparison correction	<0.0001	0.0001	***
Fig. 5H	Cartilage CS fluorescence intensity	NT-CIA vs NT-blank	One-way ANOVA	Holm–Šídák multiple-comparison correction	0.008	0.0200	*
Fig. 5H	Cartilage CS fluorescence intensity	HT-CIA vs HT-blank	One-way ANOVA	Holm–Šídák multiple-comparison correction	0.0020	0.0060	**
Fig. 5H	Cartilage CS fluorescence intensity	HT-CIA vs NT-CIA	One-way ANOVA	Holm–Šídák multiple-comparison correction	0.0400	0.0400	*
Fig. 6E	Arthritis score, Day 56	NT-CIA+CS vs NT-CIA	Two-way ANOVA	Holm–Šídák multiple-comparison correction	0.0213	0.0430	*
Fig. 6E	Arthritis score, Day 56	HT-CIA+CS vs HT-CIA	Two-way ANOVA	Holm–Šídák multiple-comparison correction	0.0431	0.0431	*
Fig. 6H	Serum IL-6	NT-CIA+CS vs NT-CIA	One-way ANOVA	Holm–Šídák multiple-comparison correction	0.0900	0.0900	ns
Fig. 6H	Serum IL-6	HT-CIA+CS vs HT-CIA	One-way ANOVA	Holm–Šídák multiple-comparison correction	0.0030	0.0070	**
Fig. 6I	Serum IL-17A	NT-CIA+CS vs NT-CIA	One-way ANOVA	Holm–Šídák multiple-comparison correction	0.0500	0.0500	*
Fig. 6I	Serum IL-17A	HT-CIA+CS vs HT-CIA	One-way ANOVA	Holm–Šídák multiple-comparison correction	0.0020	0.0050	**
Fig. 6L	Cartilage CS fluorescence intensity	NT-CIA+CS vs NT-CIA	One-way ANOVA	Holm–Šídák multiple-comparison correction	0.0020	0.0020	**
Fig. 6L	Cartilage CS fluorescence intensity	HT-CIA+CS vs HT-CIA	One-way ANOVA	Holm–Šídák multiple-comparison correction	<0.0010	<0.0010	***
Fig. 7C	Cartilage CS fluorescence intensity	NT-CIA+*P. stercorea* vs NT-CIA	One-way ANOVA	Holm–Šídák multiple-comparison correction	0.0156	0.0460	*

Figure panel	Outcome/parameter	Comparison	Statistical test	Correction method	Raw *p* value	Adjusted *p* value	Significance after correction
Fig. 7C	Cartilage CS fluorescence intensity	HT-CIA+*P. stercorea* vs HT-CIA	One-way ANOVA	Holm–Šídák multiple-comparison correction	0.8779	0.8779	ns
Fig. 8E	Arthritis score, Day 56	CIA+CS+*P. stercorea* vs CIA+*P. stercorea*	One-way ANOVA	Holm–Šídák multiple-comparison correction	<0.0010	0.0020	**
Fig. 8E	Arthritis score, Day 56	CIA+CS+*P. stercorea* vs CIA+CS	One-way ANOVA	Holm–Šídák multiple-comparison correction	0.4300	0.4300	ns
Fig. 8H	Serum IL-6	CIA+CS+*P. stercorea* vs CIA+*P. stercorea*	One-way ANOVA	Holm–Šídák multiple-comparison correction	<0.0010	<0.0010	***
Fig. 8H	Serum IL-6	CIA+CS+*P. stercorea* vs CIA+CS	One-way ANOVA	Holm–Šídák multiple-comparison correction	0.1500	0.1500	ns
Fig. 8I	Serum IL-17A	CIA+CS+*P. stercorea* vs CIA+*P. stercorea*	One-way ANOVA	Holm–Šídák multiple-comparison correction	0.0100	0.0400	*
Fig. 8I	Serum IL-17A	CIA+CS+*P. stercorea* vs CIA+CS	One-way ANOVA	Holm–Šídák multiple-comparison correction	0.0040	0.0100	*
Fig. 8L	Cartilage CS fluorescence intensity	CIA+CS+*P. stercorea* vs CIA+*P. stercorea*	One-way ANOVA	Holm–Šídák multiple-comparison correction	0.2200	0.6400	ns
Fig. 8L	Cartilage CS fluorescence intensity	CIA+CS+*P. stercorea* vs CIA+CS	One-way ANOVA	Holm–Šídák multiple-comparison correction	0.8400	0.9400	ns

Note: Only major prespecified comparisons directly related to the main experimental questions are listed. All possible pairwise comparisons were not included to avoid overinterpretation and to maintain focus on the primary conclusions. Adjusted p values were calculated using Holm–Šídák multiple-comparison correction where applicable. When applicable, time points are indicated in the Outcome/parameter column.
